# OTUB2 contributes to vascular calcification in chronic kidney disease via the YAP-mediated transcription of PFKFB3

**DOI:** 10.7150/thno.98660

**Published:** 2025-01-01

**Authors:** Yalan Li, Xiaoyue Chen, Xueqiang Xu, Cheng Chen, Min Min, Dongmei Liang, Jiafa Ren, Huijuan Mao

**Affiliations:** 1 Department of Nephrology, Jiangsu Province Hospital, The First Affiliated Hospital of Nanjing Medical University, Nanjing Medical University, 300 Guangzhou Road, Nanjing 210029, China.; 2 Department of Medical Science, Yangzhou Polytechnic College, 458 West Wenchang Road, Yangzhou 225009, China.

**Keywords:** chronic kidney disease, vascular calcification, ubiquitin-proteasome degradation, OTUB2, YAP.

## Abstract

**Rationale:** Chronic kidney disease (CKD) is a global public health issue, with vascular calcification (VC) being a common and deadly complication. Despite its prevalence, the underlying mechanisms of VC remain unclear. In this study, we aimed to investigate whether and how Otubain-2 (OTUB2) contributes to VC.

**Methods:** The relationship between OTUB2 and VC was examined via immunohistochemical and immunofluorescence staining of discarded calcified radial arteries from uremic patients who underwent arteriovenous fistula operations. Additionally, mice were fed a 0.2% adenine diet supplemented with 1.2% phosphorus to establish a model of CKD-related VC. Vascular smooth muscle cell (VSMC)-specific OTUB2 knockout and overexpression were performed *in vivo* via the delivery of adeno-associated virus 9 vectors to manipulate the expression of OTUB2. Additionally, a calcified VSMC model was established to explore the roles of OTUB2 in VC by evaluating changes in osteogenic marker expression and calcium deposition.

**Results:** Our results revealed a significant upregulation of OTUB2 expression during VC progression. OTUB2 overexpression upregulated the expression of osteogenic markers and exacerbated VSMC calcification, as verified by Von Kossa and Alizarin red staining. Conversely, VSMC-specific OTUB2 deficiency significantly mitigated adenine diet-induced VC in CKD mice. OTUB2 knockdown or inhibition decreased Yes-associated protein (YAP) abundance. Mechanistically, OTUB2 bound to YAP, decreasing its K48-linked polyubiquitination and inhibiting its subsequent degradation. Knockdown or inhibition of YAP abolished the effect of OTUB2 overexpression on VSMC calcification, indicating a YAP-mediated mechanism. Furthermore, the YAP/TEAD1 complex bound to the promoter of PFKFB3, increasing its transcriptional activity, as determined by CUT&RUN-qPCR. The knockdown or inhibition of PFKFB3 alleviated the procalcific effects of OTUB2.

**Conclusions:** Our findings indicate that OTUB2 promotes VC at least partially by activating the YAP-PFKFB3 signaling pathway. Targeting OTUB2 may be an appealing therapeutic strategy for VC.

## Introduction

The poor prognosis of chronic kidney disease (CKD) patients is largely due to vascular calcification (VC), which is an independent risk factor for CKD-related mortality 1. VC is characterized by the abnormal deposition of crystalline hydroxyapatite in the vasculature 2. The prevalence rate of VC in patients with end-stage renal disease (ESRD) undergoing dialysis is 90.7%, with 86.5% of patients experiencing VC progression over a 4-year follow-up 3. VC is closely related to decreased vascular compliance and increased stiffness, and it is an important promoter of adverse cardiovascular events such as heart failure, hypertension, and arrhythmia 4-5. Currently, traditional therapies for severe VC, such as adequate dialysis and phosphorus reduction measures, show limited efficacy and fail to retard the progression of VC. Therefore, further investigations to elucidate the molecular mechanisms of VC are urgently needed.

VC is an active process regulated by multiple cell types, including vascular smooth muscle cells (VSMCs), macrophages, and endothelial cells 6-7. The osteogenic reprogramming of VSMCs, the predominant cells in the middle layer of blood vessels, is a key process in VC, and this reprogramming is characterized by the loss of the contractile phenotype and the acquisition of the osteogenic phenotype in VSMCs 8. VC is a complex phenomenon involving multiple molecular mechanisms, such as aberrant epigenetic activation, disorders of calcium and phosphate metabolism, calcium deposition in the vasculature, mitochondrial dysfunction, and anomalous activation of transcription factors 9-11. Yes-associated protein (YAP), a crucial component of the Hippo signaling pathway, has been widely studied in cardiovascular disorders because of its critical roles in cell differentiation, proliferation, metabolism, and apoptosis 12. Multiple studies have suggested that YAP functions as a molecular switch in the development of VC 13-14. Moreover, hyperactivation of YAP has been observed in the aortas of CKD model mice compared with those of sham mice 15. The activity and expression of YAP can be finely tuned by posttranslational modifications, such as ubiquitination, phosphorylation, and methylation 16-18. Additionally, several findings indicate an intimate link between protein ubiquitination abnormalities and VC 19-20.

Ubiquitination involves the covalent attachment of small ubiquitin (Ub) proteins to substrate proteins, regulating their activity, stability, and subcellular localization 21. Ubiquitination can be reversed by deubiquitinases (DUBs) 22. Otubain-2 (OTUB2), a member of the ovarian tumor-like protease (OTU) family, regulates a variety of biological processes, including cell proliferation, DNA damage repair, and cell differentiation 23-25. Prior studies have substantiated the importance of OTUB2 in the osteogenic transdifferentiation of mesenchymal stem cells (MSCs) 25. Although the molecular biology of this protein has been extensively studied, no reports have described the potential role of OTUB2 in VC. Given that VC shares similarities with bone formation, we hypothesized that OTUB2 plays a crucial role in VC.

Increasing evidence suggests an indispensable role for glycolytic metabolism in VC, with multiple glycolytic enzymes being upregulated in calcified VSMCs 11, 26. The role of 6-phosphofructo-2-kinase/fructose-2,6-bisphosphatase isoform 3 (PFKFB3) in VC is increasingly recognized. Niu J et al. reported that the κ-opioid receptor inhibits VSMC calcification by downregulating PFKFB3 expression 27. PFKFB3 drives glycolysis in VSMCs and promotes VC by increasing lactate production 28. In the present study, we found that OTUB2-mediated YAP stabilization promotes PFKFB3 transcription, thereby accelerating VC in CKD. Our study aimed to elucidate the OTUB2-mediated epigenome-metabolome signaling cascade and provide potential therapeutic targets for VC.

## Materials and Methods

### Patient sample collection

All the related procedures for collecting radial arteries from CKD patients were performed in strict accordance with the Declaration of Helsinki. This study was approved by the Human Research Ethics Committee of the First Affiliated Hospital of Nanjing Medical University (approval number: 2024-SR-252). All patients were adequately informed and provided written consent. The discarded radial arteries were obtained from uremic patients who underwent arteriovenous fistula surgery and were subsequently embedded in wax blocks. The basic information of the patients was obtained from the electronic medical records available at the hospital.

### Induction of VC in mice

C57BL/6J mice, aged six to eight weeks, were purchased from the Laboratory Animal Center of Nanjing Medical University. Male mice were used to avoid any possible effects of different hormone levels on VC. All experiments were approved by the Institutional Animal Care Committee of Nanjing Medical University, China (IACUC-2212041), and performed in accordance with the US National Institutes of Health Guide for the Care and Use of Laboratory Animals. All animals were raised in a specific pathogen-free (SPF) environment and maintained in a temperature-controlled room with a 12-h light/dark cycle. The animals were randomly assigned to different groups, with a minimum of six mice per group.

The control group was fed a standard pellet chow diet (normal diet, ND), and the CKD model group was fed special chow containing 0.2% adenine and a high concentration (1.2%) of phosphorus (AP diet). To increase the palatability and odor of the adenine-containing diet, 6% casein was added. For VSMC-specific OTUB2 overexpression or knockdown, mice were injected via the lateral tail vein with recombinant adeno-associated virus 9 (AAV9) vectors bearing a VSMC-specific promoter combination (SM22α promoter) with a mouse OTUB2 OE or shOTUB2 sequence at a dose of 10^11^ v.g per mouse (Hanbio, China). After 12 weeks on the AP diet, the mice were anesthetized with 2.5% isoflurane, euthanized via an intravenous injection of pentobarbital sodium. The blood and aortas were collected and stored at -80 °C until further use.

### Cell culture

Mouse VSMCs were isolated from the aorta via the explant method as previously described 29. Eight-week-old male C57BL/6J mice weighing 20 to 25 g were purchased from the Laboratory Animal Center of Nanjing Medical University, China. Briefly, the mice were euthanized, and their thoracic aortas were dissected. The adventitia and endothelium were removed from the thoracic aortic arteries, and the remaining tissue was cut into approximately 1 mm^2^ sections under a gross microscope. Cells between passages 3 and 8 were used in this study and cultured in growth medium (GM), which was composed of DMEM/F12 (Gibco, USA) supplemented with 10% fetal bovine serum (FBS; Sciencell, USA), and 1% penicillin/streptomycin (KeyGEN, China). The cells were treated with calcifying medium (CM) for 7 days to induce VSMC calcification. The CM consisted of GM supplemented with 10 mM β-glycerophosphate (β-GP; Solarbio, China) and 0.05 mg/mL ascorbic acid (Solarbio, China). HEK293T cells obtained from ATCC were cultured in DMEM supplemented with 10% FBS at 37 °C with 5% CO_2_. All media were changed every second day.

### Cell transfection

To knock down endogenous protein expression, VSMCs were seeded in 6-well plates. Upon reaching 60-70% confluence, VSMCs were transfected with empty vector or specific small interfering RNA (siRNA) against OTUB2, YAP, or PFKFB3 (Hippobiotec, China) using Lipofectamine 8000 transfection reagent (Beyotime, China) in Opti-MEM according to the manufacturer's instructions. After transfection for 6-8 h at 37 °C, the media were replaced with fresh GM, and the cells were used for further experiments. The sequences of the siRNA oligos used are listed in supplementary Table S1.

### Western blot analysis

Proteins were extracted from VSMCs and vascular tissues using cold RIPA lysis buffer (FDbio, China) containing proteinase and phosphatase inhibitors (FDbio, China). The cell lysates were collected in 1.5 mL tubes and centrifuged at 12,000 rpm for 15 min. Samples were subjected to SDS-PAGE, transferred to polyvinylidene difluoride (PVDF) membranes (Millipore, USA), and blocked with 5% bovine serum albumin (Beyotime, China) for at least 1 h at room temperature to eliminate nonspecific binding of the antibodies. After incubation with the primary antibodies against the target proteins overnight at 4 °C, the membranes were washed three times and incubated with secondary antibodies (FDbio, China) for 1 h at room temperature. The blots were visualized using chemiluminescence reagents and quantified with ImageJ software (NIH, USA). The protein level was normalized to that of GAPDH, β-actin, or α-tubulin and expressed as the relative change compared with the control. The primary antibodies used in this study are listed in supplementary Table S2.

### Quantitative real-time polymerase chain reaction

Total RNA was extracted from incubated VSMCs using an RNA extraction kit (Vazyme, China), and the concentration of RNA was measured via a spectrophotometer. The extracted RNA was reverse-transcribed into cDNA with a PrimeScript RT reagent kit (Vazyme, China) according to the manufacturer's instructions. Gene expression levels were measured using real-time PCR (Vazyme, Nanjing) with a Roche real-time PCR system (LightCycler 96). Relative mRNA expression levels were analyzed using the 2^-ΔΔCt^ method and normalized to that of β-actin or GAPDH. The primer sequences are listed in supplementary Table S3.

### Alizarin red and Von Kossa staining

Alizarin red and Von Kossa staining were performed to assess calcification in the VSMCs and aortas. Briefly, VSMCs were fixed with 4% paraformaldehyde (PFA; Biosharp, China) for 15 min and subsequently rinsed with deionized water (ddH_2_O). For vascular tissue samples, paraffin-embedded sections were subjected to routine deparaffinization and rehydration. A silver nitrate (AgNO_3_) solution (Solarbio, China) was applied to the sections or VSMCs, which were then exposed to ultraviolet light for 2 h. Following three washes with ddH_2_O, the sections or VSMCs were then incubated with sodium thiosulfate for 5 min. The calcified spots were stained black. For Alizarin red staining, VSMCs or histological sections were incubated with 2% Alizarin red solution (pH 4.2, Solarbio, China) for 30 min in the dark. After washing with ddH_2_O, positively stained areas appeared orange-red.

### Serum analyses

Blood was collected from the eyes of the mice and centrifuged at 3000 rpm for 15 min. The supernatants were stored at -80 °C. Serum levels of phosphorus (Pi), calcium (Ca), creatinine (Cr), and blood urea nitrogen (BUN) were determined using commercial kits from the Nanjing Jiancheng Institute.

### Immunofluorescence staining

Mouse aortas were fixed overnight with 4% PFA and embedded in paraffin. The tissues were sectioned at 5-μm thickness and dewaxed according to standard protocols. After heat-mediated antigen retrieval, the sections were blocked with a sealing solution containing 10% goat serum (KeyGEN, China) for 1 h at room temperature and incubated with primary antibody overnight at 4 °C. The next day, the sections were washed with PBST three times and stained with the corresponding secondary antibodies (Abways, China) for 1 h at room temperature. Finally, the nuclei were counterstained with 4',6'-diamidino-2-phenylindole (DAPI; Beyotime, China), and images were acquired under a THUNDER DMi8 microscope (Leica, Germany).

### Immunohistochemical staining

Aortic sections were dewaxed, hydrated, and subjected to heat-mediated antigen retrieval using routine protocols. The sections were subsequently washed and blocked with 10% donkey serum (1 h, room temperature). After incubation with diluted primary antibodies overnight at 4 °C, the sections were incubated with secondary antibodies (MXB, China) for 1 h at 37 °C. After three washes with PBST, the DAB staining working solution was prepared according to the instructions. Regions of positive immunostaining appeared as a brown color. Hematoxylin (KeyGEN, China) counterstaining was performed, and after thorough rinses with tap water, the tissues were observed and imaged.

### Co-immunoprecipitation

For Co-immunoprecipitation (Co-IP), the experimental procedures were performed in accordance with the instructions of the Co-IP kit provided by Suzhou Beaver Bioengineering Company. Briefly, VSMCs or HEK293T cells were collected and lysed with precooled IP lysis buffer (Absin, China). The protein lysates were incubated with primary antibodies overnight at 4 °C. Anti-IgG antibodies were used as controls. Protein A/G beads were then added to the antibody-antigen complex and incubated at room temperature for 2 h. Proteins were eluted with SDS buffer and analyzed by Western blot.

### CUT&RUN-qPCR Assays

CUT&RUN-qPCR assays were performed using a CUT&RUN-qPCR assay kit (Vazyme, China) following the instruction manual. Briefly, VSMCs were collected in EP tubes, centrifuged at low speed, and resuspended in wash buffer. The cells were incubated with ConA Beads Pro at room temperature for 10 min, and the supernatant was discarded. After overnight incubation with prechilled antibody buffer, the samples were mixed with pG-MNase enzyme for 1 h. After centrifugation, DNA was fragmented with CaCl_2_ for 4-6 h at room temperature, and fragmentation was stopped by the addition of stop buffer. Finally, DNA was extracted, and qPCR was used for analysis.

### Seahorse assays

Glycolysis was assessed using a glycolytic rate assay kit (Agilent Technologies, 103344-100) and a Seahorse XF flux analyzer 96 according to the manufacturer's instructions. Briefly, VSMCs were seeded on Seahorse XF-96 plates at a density of 1.5×10^4^ cells/well. The probe plate was hydrated in a CO_2_-free incubator, the VSMC culture medium was replaced with phenol red-free assay solution, and the plate was incubated at 37 °C in a non-CO_2_ incubator for 1 h to measure the glycolytic proton efflux rate (glycoPER). Next, Rot/AA (mitochondrial electron transport chain inhibitor, 0.5 μM) and 2-DG (2-deoxyd-glucose, 50 μM) were injected according to the manufacturer's protocols. The experimental data were analyzed using Wave version 2.2 software.

### Statistical analysis

Statistical analyses of all the data were performed using GraphPad Prism, version 9.0. The results were presented as the mean ± standard deviation (SD). All experiments were independently repeated at least three times. Data normality was tested using the Shapiro-Wilk test. Student's t test was performed for comparisons between two groups. One-way ANOVA was used to compare the differences between multiple groups. *P* < 0.05 was considered statistically significant.

## Results

### OTUB2 expression is upregulated during VC

**Figure 1:**
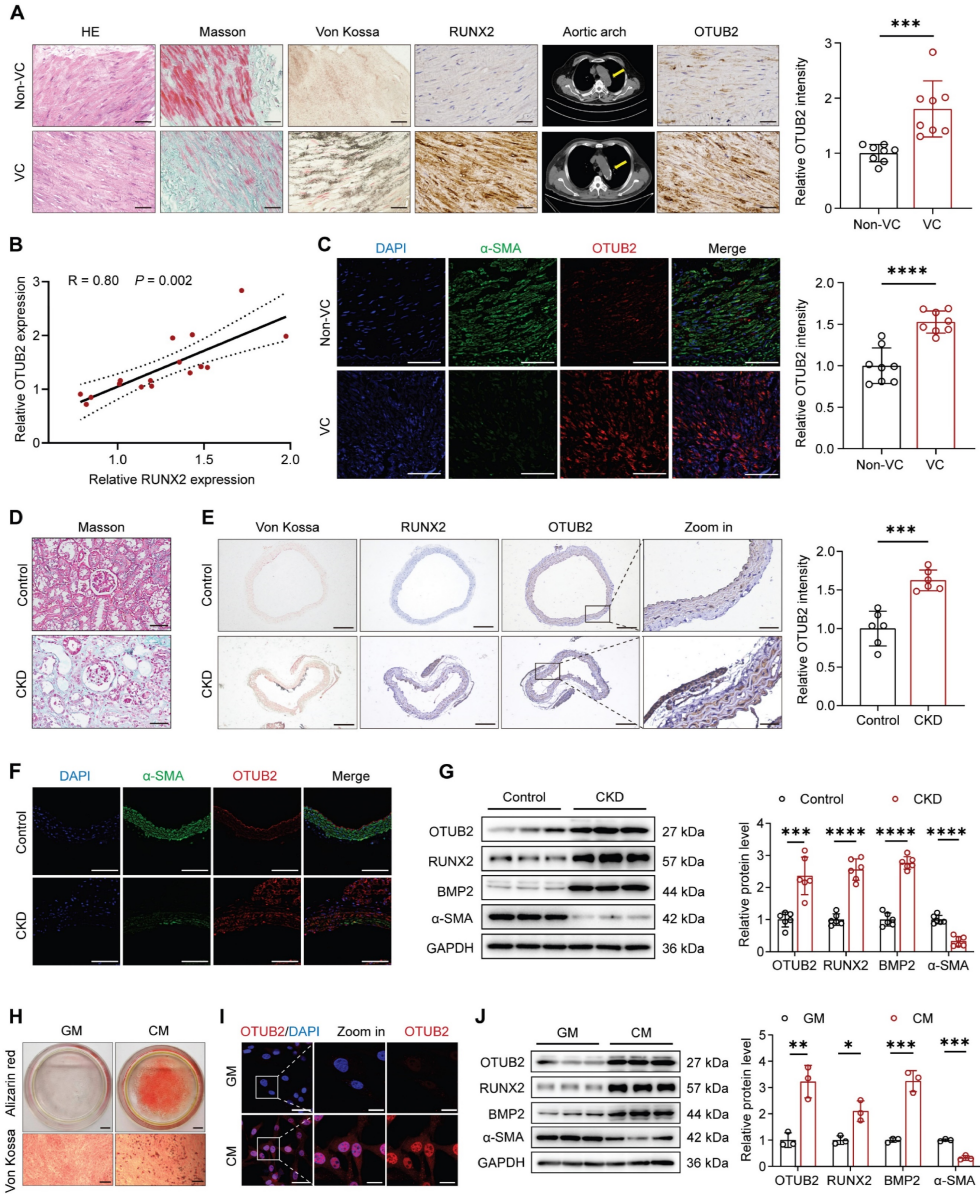
** OTUB2 expression is upregulated during VC. (A)** Representative images of HE staining, Masson's trichrome staining, Von Kossa staining, and immunohistochemical staining for RUNX2 and OTUB2 expression in radial arteries and multidetector computed tomography (MDCT) scans of CKD patients without or with calcification. Scale bars, 50 μm. n = 8 per group. **(B)** Scatter dot plot of the correlation between OTUB2 and RUNX2 expression in radial arteries from CKD patients without or with calcification. n = 16. **(C)** Representative images of immunofluorescence staining for OTUB2 and α-SMA in radial arteries from CKD patients without or with calcification. Scale bars, 100 μm. n = 8 per group. **(D)** Representative images of Masson's trichrome staining of kidneys from the control and CKD groups. Scale bars, 50 μm. **(E)** Representative images of Von Kossa staining and immunohistochemical staining for RUNX2 and OTUB2 in the arteries of mice from the control and CKD groups. Scale bars, 200 μm (left panels), 50 μm (right panels). n = 6 per group. **(F)** Representative images of immunofluorescence staining for OTUB2 and α-SMA in the arteries of the indicated groups. Scale bars, 100 μm. **(G)** Western blot analysis and quantification of OTUB2, RUNX2, BMP2, and α-SMA expression. n = 6 per group. **(H)** Representative images of Alizarin red and Von Kossa staining. Scale bars, 5 mm (upper panels), 100 μm (lower panels). **(I)** Representative images of immunofluorescence staining for OTUB2 expression in VSMCs treated with GM or CM. Scale bars, 50 μm (left panels), 20 μm (right panels). **(J)** Immunoblots and quantification of OTUB2, RUNX2, BMP2, and α-SMA protein expression in VSMCs treated with GM or CM. n = 3 per group. Statistical significance was assessed using two-tailed t-test **(A, C, E, G, J)**. All values are presented as mean ± SD. *P < 0.05, **P < 0.01, ***P < 0.001, and ****P < 0.0001.

To determine whether an association exists between OTUB2 and arterial calcification, radial arteries from CKD patients who underwent arteriovenous fistula surgery were used to detect OTUB2 expression. The participants were divided into noncalcified and calcified groups based on the results of chest multidetector computed tomography (MDCT) scans of the aorta. The characteristics of the CKD patients in the non-VC and VC groups are shown in supplementary Table S4. Morphological changes were observed via hematoxylin and eosin (HE) staining, and collagen deposition in the radial arteries was assessed via Masson's trichrome staining. Von Kossa staining, immunohistochemical (IHC) staining for RUNX2, and MDCT scans were performed to verify VC (Figure 1A and Figure S1A). Compared with noncalcified arteries, calcified arteries presented an apparent increase in collagen and calcium deposition and a significant upregulation of RUNX2 expression (Figure 1A). We found that the levels of OTUB2 were markedly upregulated in calcified samples (Figure 1A). In addition, the protein expression level of OTUB2 was positively correlated with the expression of the osteogenic marker RUNX2 (Figure 1B). Compared with non-VC samples, calcified samples presented elevated OTUB2 expression in parallel with a reduction in contractile marker (α-SMA) expression (Figure 1C).

Furthermore, we established a CKD model by feeding mice a diet containing 0.2% adenine for 12 weeks and collected the serum after euthanasia. Compared with control mice, CKD model mice presented obvious weight loss, and the Cr, BUN, and Pi levels were significantly increased. The serum Ca levels did not differ significantly between the two groups (Table S5). Additionally, Western blot analysis and renal histological staining revealed interstitial fibrosis, tubular atrophy, and calcium deposition in small vessels (Figure 1D and Figure S1B-C). Von Kossa staining revealed more positive staining in the aortas of the CKD group than those of the control group (Figure 1E and Figure S1D). These data revealed the successful establishment of the CKD model. IHC and immunofluorescence (IF) staining revealed that significantly higher OTUB2 expression in the calcified aortas of the CKD group than in those of the control group (Figure 1E-F). Western blot assays further confirmed the increased OTUB2 protein levels in the aortas of the CKD group (Figure 1G). These results underscore the relevance of OTUB2 in CKD-associated VC.

We then explored the effects of the VSMC osteogenic transdifferentiation on OTUB2 expression. Primary VSMCs were extracted using the organizational patch method, and their purity was confirmed by immunostaining for α-SMA (Figure S1E). The calcification of VSMCs was successfully induced by CM, as evidenced by Alizarin red and Von Kossa staining (Figure 1H). IF staining revealed that OTUB2 protein expression increased in response to CM stimulation (Figure 1I). Moreover, RT-qPCR and Western blot assays revealed that CM significantly increased the mRNA and protein levels of OTUB2 (Figure 1J and Figure S1F). Additionally, Otubain-1 (OTUB1) and OTUB2 belong to the same subfamily, which was previously reported to play important roles in the regulation of bone homeostasis and atherosclerosis 30-31. Therefore, we also examined the changes in OTUB1 expression during VSMC osteogenic differentiation. Notably, the level of OTUB1 did not change appreciably (Figure S1F-G). Collectively, these results demonstrated that OTUB2 expression is upregulated in VC.

### OTUB2 accelerates the development of VC

**Figure 2:**
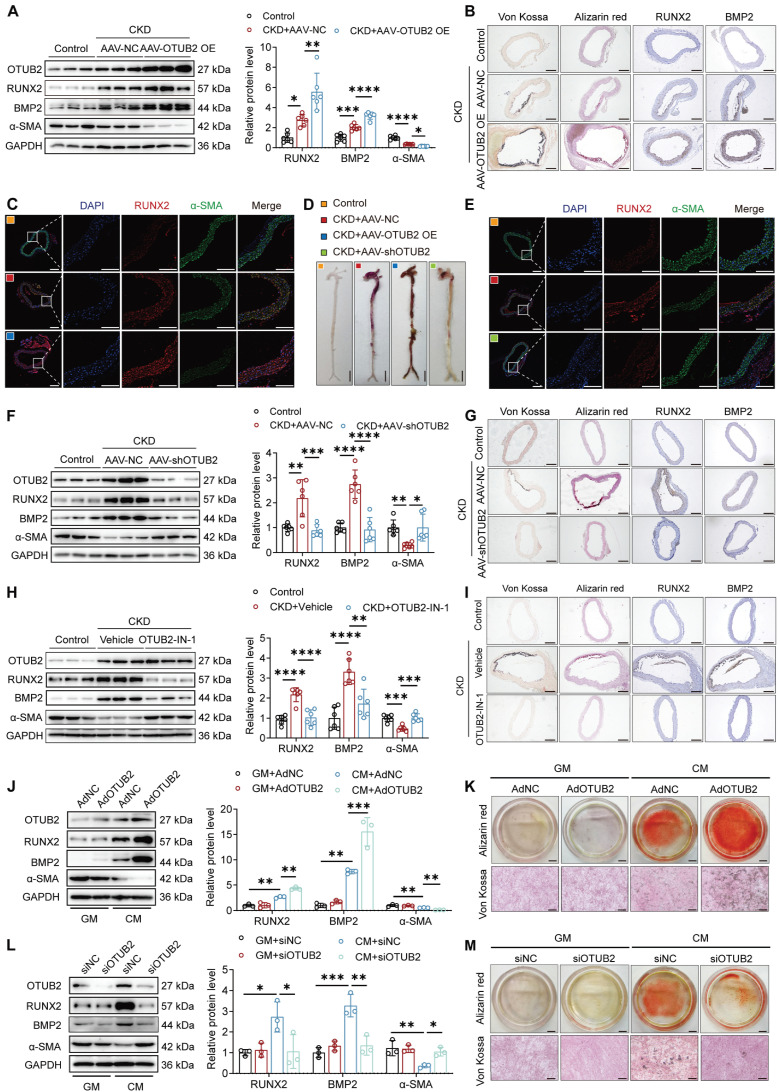
** OTUB2 accelerates the development of VC. (A)** Western blot analysis of OTUB2, RUNX2, BMP2 and α-SMA expression in aortas. n = 6 per group. **(B)** Representative images of Von Kossa staining, Alizarin red staining, and immunohistochemical staining for RUNX2 and BMP2 in aortas from the indicated experimental cohorts. Scale bars, 200 μm. **(C)** Representative images of immunofluorescence staining for RUNX2 and α-SMA in aortas from the different experimental groups. Scale bars, 200 μm (left panels), 100 μm (right panels). **(D)** Representative images of Alizarin red staining of whole aortas from the indicated experimental groups. Scale bars, 5 mm. **(E)** Representative images of immunofluorescence staining for RUNX2 and α-SMA in aortas from the different experimental groups. Scale bars, 200 μm (left panels), 100 μm (right panels). **(F)** Western blot analysis of OTUB2, RUNX2, BMP2 and α-SMA expression in aortas. n = 6 per group. **(G)** Representative images of Von Kossa staining, Alizarin red staining, and immunohistochemical staining for RUNX2 and BMP2 in aortas from the indicated experimental cohorts. Scale bars, 200 μm. **(H)** Western blot analysis of OTUB2, RUNX2, BMP2 and α-SMA expression in aortas. n = 6 per group. **(I)** Representative images of Von Kossa staining, Alizarin red staining, and immunohistochemical staining for RUNX2 and BMP2 in aortas from the indicated experimental cohorts. Scale bars, 200 μm. **(J)** Western blot analysis of OTUB2, RUNX2, BMP2, and α-SMA expression in VSMCs overexpressing OTUB2. n = 3 per group. **(K)** Representative images of Alizarin red and Von Kossa staining of VSMCs after transfection of the indicated constructs and CM exposure for another 7 days. Scale bars, 5 mm (upper panels), 100 μm (lower panels). **(L)** Western blot analysis of OTUB2, RUNX2, BMP2, and α-SMA expression in VSMCs with OTUB2 depletion. n = 3 per group. **(M)** Representative images of Alizarin red and Von Kossa staining of VSMCs after the transfection of the indicated constructs and CM exposure for another 7 days. Scale bars, 5 mm (upper panels), 100 μm (lower panels). Statistical significance was assessed using one-way ANOVA followed by Dunnett's test **(A, F, H, J and L)**. All values are presented as mean ± SD. *P < 0.05, **P < 0.01, ***P < 0.001, and ****P < 0.0001.

Given that OTUB2 is upregulated during VC development, we next sought to evaluate whether OTUB2 overexpression might accelerate VC. We used a recombinant adeno-associated virus (AAV) carrying an empty vector (AAV-NC) or OTUB2 (AAV-OTUB2 OE) to manipulate OTUB2 expression *in vivo* (Figure S2A). The overexpression efficiency of OTUB2 was confirmed by Western blot and immunohistochemical staining, and OTUB2 overexpression did not affect metabolic parameters in CKD model mice (Figure S2B-E and Table S5). Compared with those in the AAV-NC group, the expression of osteogenic markers (RUNX2 and BMP2) was further increased, and the expression of a contractile marker (α-SMA) was further decreased by OTUB2 overexpression (Figure 2A-C). Consistently, increased calcium deposition was observed in the aortas of the AAV-OTUB2 OE mice compared with those of the AAV-NC mice (Figure 2D and Figure S2F).

We injected an AAV carrying a scrambled shRNA or OTUB2 shRNA (shOTUB2) into CKD model mice via the tail vein to further determine the role of OTUB2 in VC (Figure S2G). OTUB2 was successfully knocked down (Figure S2E, S2H-I). No significant differences in body weight or metabolic parameters were observed between AAV-shOTUB2 mice and AAV-NC mice (Table S5). The results indicated that calcium deposition and VSMC osteogenic differentiation were obviously reduced in the aortas of AAV-shOTUB2 mice, accompanied by a significant decrease in the plasma alkaline phosphatase (ALP) level, which is known to be associated with an increased occurrence of VC (Figure 2D-G and Figure S2J). We also injected mice intraperitoneally with OTUB2-IN-1, a specific inhibitor of OTUB2, which was recently identified as able to block the deubiquitinase activity of OTUB2 but did not affect its stability (Figure S2K) 32. Notably, the administration of OTUB2-IN-1 did not affect metabolic parameters in CKD model mice (Table S6). OTUB2-IN-1 treatment markedly reduced the calcified area and suppressed VSMC osteogenic differentiation in the aortas of CKD model mice (Figure 2H-I and Figure S2L-P). Collectively, these findings suggest that OTUB2 exacerbates the severity of VC and that OTUB2 deficiency and inhibition may be effective therapeutic strategies for VC.

### OTUB2 regulates the osteogenic differentiation of VSMCs

We next assessed the effects of OTUB2 on the osteogenic transdifferentiation of VSMCs *in vitro.* We performed OTUB2 overexpression and knockdown in VSMCs with recombinant adenoviruses and siRNAs, and the transfection efficiency was verified by Western blot (Figure S3A-B). Compared with the CM+AdNC group, the CM+AdOTUB2 group presented further increases in osteogenic marker (RUNX2 and BMP2) expression and decreases in contractile marker (α-SMA) expression, together with increased calcium deposition (Figure 2J-K). In contrast, OTUB2 deficiency dramatically suppressed VSMC calcification and decreased the expression of RUNX2 and BMP2, and, conversely, increased the expression of α-SMA (Figure 2L-M). In addition, OTUB2 inhibition by OTUB2-IN-1 attenuated calcium deposition and osteogenic reprogramming in VSMCs compared with VSMCs treated with the vehicle (Figure S3C-D). Thus, these data suggest that OTUB2 promotes the osteogenic differentiation of VSMCs.

### OTUB2 activates YAP in VSMCs

**Figure 3:**
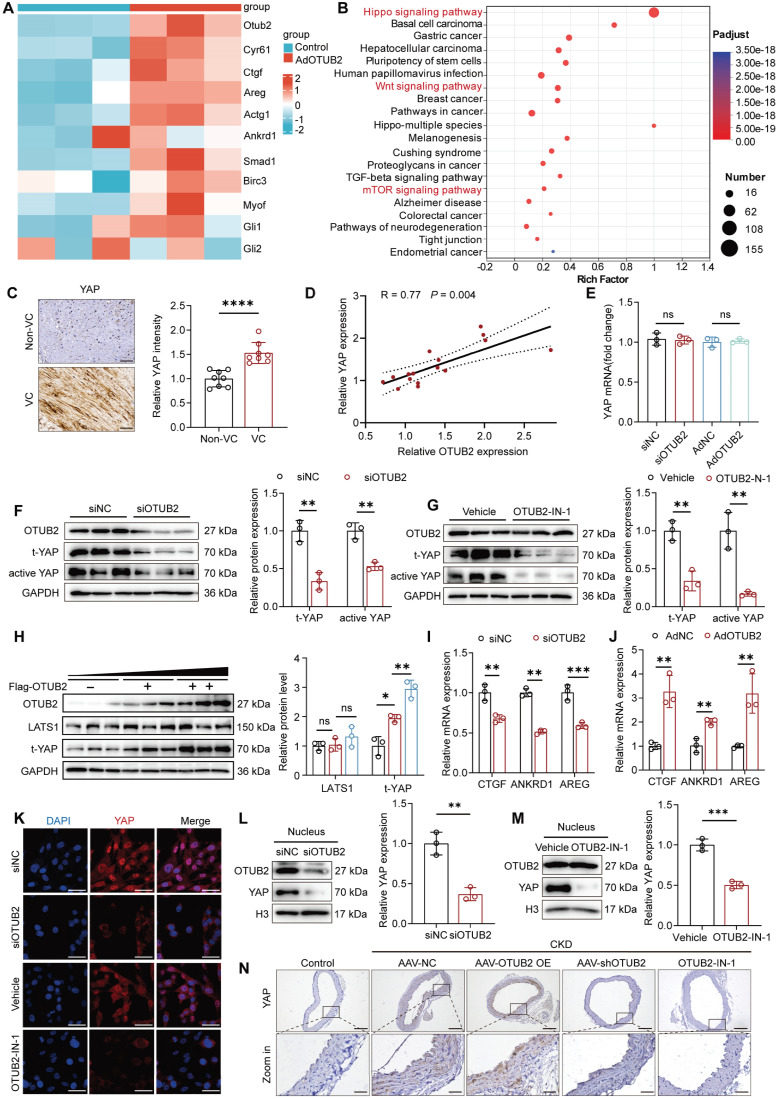
** OTUB2 activates YAP in VSMCs. (A)** Heatmap of differentially expressed YAP-targeted genes identified using RNA-seq data from VSMCs treated with control adenoviruses or adenoviruses overexpressing OTUB2. **(B)** KEGG enrichment analysis of the differentially expressed genes in OTUB2-overexpressing VSMCs vs. control VSMCs. **(C)** Representative images of immunohistochemical staining for YAP in radial arteries from CKD patients. Scale bars, 50 μm. n = 8 per group. **(D)** Scatter dot plot of the correlation between YAP and OTUB2 expression in radial arteries from CKD patients. n = 16. **(E)** RT-qPCR analysis of YAP expression after the transfection of the indicated constructs. n = 3 per group. **(F)** Western blot analysis and quantification of total and active YAP levels in VSMCs with OTUB2 depletion. n = 3 per group. **(G)** Western blot analysis and quantification of total and active YAP levels in VSMCs with OTUB2 inhibition by OTUB2-IN-1. n = 3 per group. **(H)** Western blot analysis and quantification of YAP and LATS1 levels in VSMCs. n = 3 per group. **(I)** RT-qPCR analysis of YAP target gene expression under OTUB2 depletion conditions. n = 3 per group. **(J)** RT-qPCR analysis of YAP target gene expression under OTUB2-overexpressing conditions. n = 3 per group. **(K)** Immunofluorescence staining revealed that OTUB2 silencing and inhibition suppressed YAP nuclear translocation. Scale bars, 50 μm. (**L-M**) Nucleocytoplasmic separation assays revealed that OTUB2 depletion and inhibition inhibited the nuclear translocation of YAP. n = 3 per group. **(N)** Immunohistochemical staining for YAP in aortic sections from the indicated groups. Scale bars, 200 μm (upper panels) and 50 μm (lower panels). n = 6 per group. Statistical significance was assessed using two-tailed t-test **(C, E, F, G, I, J, L and M)** and one-way ANOVA followed by Dunnett's test **(H)**. All values are presented as mean ± SD. *P < 0.05, **P < 0.01, ***P < 0.001, and ****P < 0.0001.

To better explore the regulatory mechanism underlying the procalcific effect of OTUB2, we performed transcriptome sequencing (RNA-seq) to evaluate transcriptional changes in VSMCs after OTUB2 overexpression. The RNA-seq results revealed that the expression of a total of 1387 genes was significantly changed in response to OTUB2 overexpression. Differentially expressed genes (DEGs) were identified with a false discovery rate < 0.05 and an absolute log2-fold change > 1.5. The volcano plot shows that, of these 1387 DEGs, 803 genes were significantly upregulated by OTUB2 overexpression, and 584 genes were downregulated (Figure S4A). The heatmap analysis revealed that OTUB2 overexpression significantly affected the expression of YAP target genes, including CTGF, CYR61, and AREG (Figure 3A). The Kyoto Encyclopedia of Genes and Genomes (KEGG) pathway analysis revealed several enriched calcification-related pathways, such as the Hippo pathway, the Wnt pathway, and the mTOR pathway (Figure 3B). We then focused on YAP, which is considered crucial for the differentiation of osteoblastic cells 33. Recent studies have shown that YAP may serve as a pivotal modulator of VC 34.

Immunohistochemical staining for YAP was conducted on a total of 8 pairs of radial arteries from CKD patients with and without VC. The findings revealed a notable increase in the level of YAP expression in the VC group compared with that in the control group (Figure 3C). Additionally, a positive correlation was observed between the expression of YAP and that of OTUB2 (Figure 3D). The expression of genes downstream of YAP was also upregulated in the aortas of CKD model mice compared with those of control mice in the GSE159832 dataset (Figure S4B). Next, we assessed the effect of OTUB2 on YAP expression. Western blot and RT-qPCR revealed that OTUB2 depletion altered the protein expression of total and active YAP but not the mRNA expression of YAP, which implied that OTUB2 had little effect on YAP transcription (Figure 3E-F). OTUB2-IN-1 was also able to reduce total and active YAP levels and did not affect the stability of OTUB2 (Figure 3G). Additionally, overexpression of OTUB2 upregulated YAP expression in a dose-dependent manner but did not affect the expression levels of upstream components of the Hippo pathway, such as LATS1, in either VSMCs or HEK293T cells (Figure 3H and Figure S4C). We examined the expression of Hippo target genes following OTUB2 overexpression or depletion and found that OTUB2 depletion inhibited the transcription of Hippo target genes, including CTGF, AREG, and ANKRD1 in VSMCs, whereas the opposite results were observed in the overexpression group (Figure 3I-J). The regulation of YAP expression by OTUB2 was also validated through an *in vivo* animal study, and the results were consistent with those of the *in vitro* experiments (Figure 3N and Figure S4D). As a transcriptional coactivator, YAP functions mainly by translocating to the nucleus 35. Furthermore, immunofluorescence staining and nucleocytoplasmic separation assays revealed that both OTUB2 knockdown and inhibition reduced YAP nuclear translocation (Figure 3K-M). These findings suggest that OTUB2 is an important regulator of YAP function.

### Knockdown or inhibition of YAP partially suppresses VC promoted by OTUB2 overexpression

**Figure 4:**
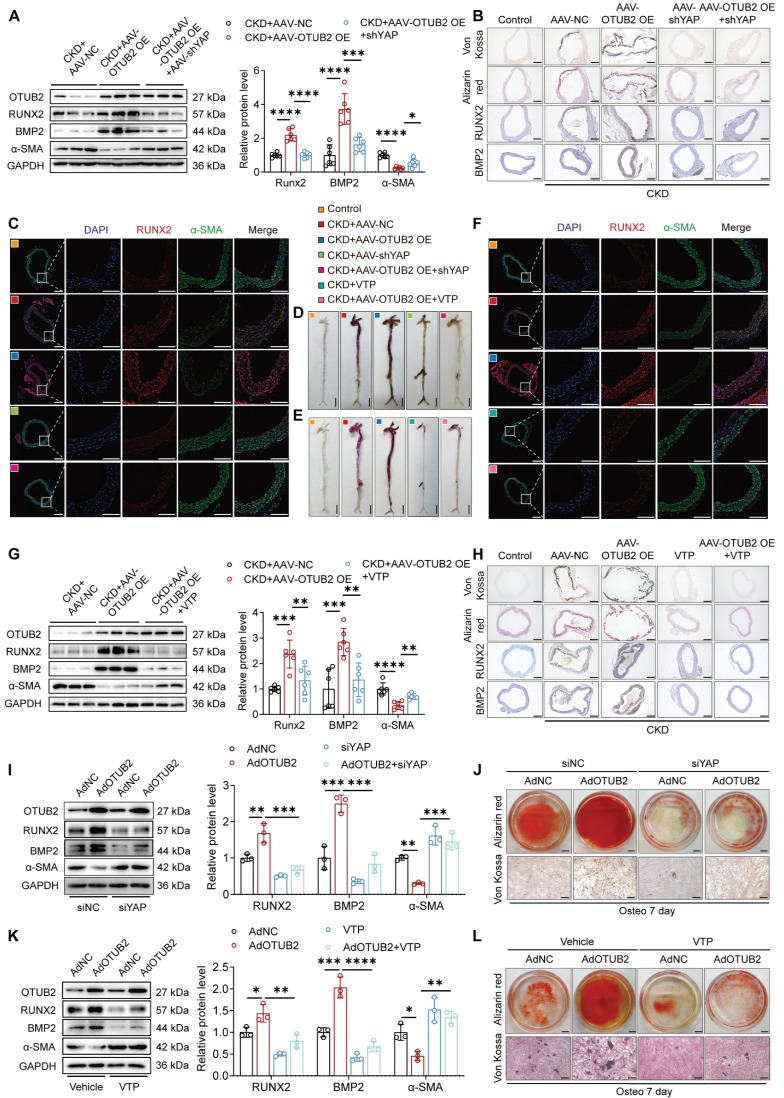
** Knockdown or inhibition of YAP partially suppresses VC promoted by OTUB2 overexpression. (A)** Western blot analysis of OTUB2, RUNX2, BMP2 and α-SMA expression in aortas. n = 6 per group. **(B)** Representative images of Von Kossa staining, Alizarin red staining, and immunohistochemical staining for RUNX2 and BMP2 in aortas from the indicated experimental cohorts. Scale bars, 200 μm. **(C)** Representative images of immunofluorescence staining for RUNX2 and α-SMA in aortas from the different experimental groups. Scale bars, 200 μm (left panels), 100 μm (right panels). (**D-E**) Representative images of Alizarin red staining of whole aortas from the different experimental groups. Scale bars, 5 mm. **(F)** Representative images of immunofluorescence staining for RUNX2 and α-SMA in aortas from the different experimental groups. Scale bars, 200 μm (left panels), 100 μm (right panels). **(G)** Western blot analysis of OTUB2, RUNX2, BMP2 and α-SMA expression in aortas. n = 6 per group. **(H)** Representative images of Von Kossa staining, Alizarin red staining, and immunohistochemical staining for RUNX2 and BMP2 in aortas from the indicated experimental cohorts. Scale bars, 200 μm. **(I)** Western blot analysis of OTUB2, RUNX2, BMP2, and α-SMA expression in VSMCs. n = 3 per group. **(J)** Representative images of Alizarin red and Von Kossa staining of VSMCs after the transfection of the indicated constructs and CM exposure for another 7 days. Scale bars, 5 mm (upper panels), 100 μm (lower panels). **(K)** Western blot analysis of OTUB2, RUNX2, BMP2, and α-SMA expression in VSMCs. n = 3 per group. **(L)** Representative images of Alizarin red and Von Kossa staining of VSMCs after the transfection of the indicated constructs and CM exposure for another 7 days. Scale bars, 5 mm (upper panels), 100 μm (lower panels). Statistical significance was assessed using one-way ANOVA followed by Dunnett's test **(A, G, I, K)**. All values are presented as mean ± SD. *P < 0.05, **P < 0.01, ***P < 0.001, and ****P < 0.0001.

To validate whether the increase in VC resulting from OTUB2 overexpression is a result of YAP signaling pathway activation, we utilized an AAV carrying a scrambled shRNA or YAP shRNA and verteporfin (VTP, a specific YAP inhibitor) to suppress YAP activity (Table S7). The effect of YAP silencing was validated *in vivo* (Figure S5A-C). Silencing of YAP significantly decreased calcium deposition in the aorta, which was increased by OTUB2 overexpression in CKD model mice (Figure 4A-D). In addition, inhibition of YAP by VTP mitigated the calcium deposition and VSMC osteogenic transdifferentiation resulting from OTUB2 overexpression (Figure 4E-H and Figure S5D).

We performed functional rescue experiments to determine whether YAP mediates OTUB2-regulated VSMC osteogenic transdifferentiation. YAP was silenced by siRNA or inhibited by VTP after the overexpression of OTUB2 in VSMCs under calcifying conditions. YAP knockdown or inhibition effectively antagonized the OTUB2-mediated increase in osteogenic differentiation (Figure 4I, 4K and Figure S5E). Alizarin red and Von Kossa staining revealed that OTUB2 overexpression promoted mineralized nodule formation in VSMCs, whereas YAP knock-down or inhibition abrogated this effect (Figure 4J, 4L). Conversely, in VSMCs cotransfected with the OTUB2 siRNA (siOTUB2) and a YAP overexpression plasmid (pcYAP), the upregulation of YAP signifi-cantly increased calcium deposition and abrogated the anticalcification effects of OTUB2 knockdown (Figure S5F-H). In summary, these results collectively suggest that the specific knockdown or suppression of YAP protects OTUB2-overexpressing VSMCs from exacerbated VC, which provides evidence of the functional role of the YAP signaling pathway in VC.

### OTUB2 interacts with and stabilizes YAP

**Figure 5:**
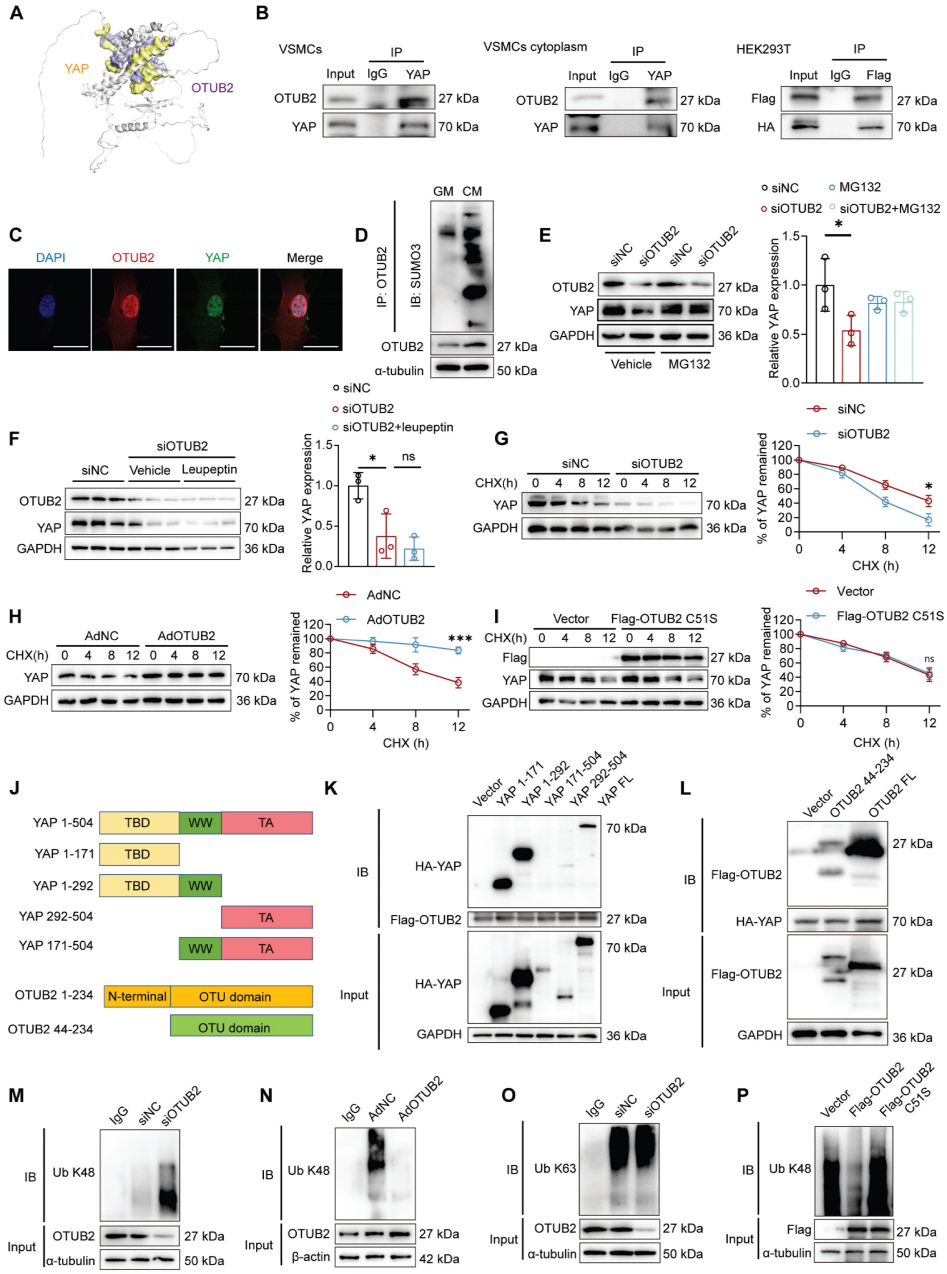
** OTUB2 interacts with and stabilizes YAP. (A)** Molecular docking assays of OTUB2 and YAP. **(B)** VSMC lysates were immunoprecipitated with an anti-YAP antibody, and HEK293T cells transfected with Flag-tagged OTUB2 and HA-tagged YAP were subjected to Co-IP assays. **(C)** Immunofluorescence staining were performed using antibodies against OTUB2 (red) and YAP (green). DAPI (blue). Scale bars, 50 μm. **(D)** SUMOylation levels of OTUB2 in VSMCs treated with GM or CM. **(E-F)** Western blot analysis and quantification of YAP expression. n = 3 per group. **(G)** Control or OTUB2-silenced VSMCs were treated with cycloheximide (CHX) and subjected to Western blot. **(H)** Control or OTUB2-overexpressing VSMCs were treated with CHX and subjected to Western blot. **(I)** An empty vector and Flag-OTUB2 C51S were transfected into VSMCs, which were then treated with CHX, and a quantitative analysis of the half-life of the YAP protein was performed. **(J)** Schematic diagram showing the wild-type and truncated YAP and OTUB2 constructs. **(K)** Representative immunoblots showing the interaction between OTUB2 and WT or truncated YAP, as indicated, as assessed by IP. **(L)** Representative immunoblots showing the interaction between YAP and WT or truncated OTUB2, as indicated, as assessed by IP. **(M)** K48-linked ubiquitination of YAP was measured by immunoblotting after OTUB2 was silenced. **(N)** K48-linked ubiquitination of YAP was measured by immunoblotting after OTUB2 was overexpressed. **(O)** K63-linked ubiquitination of YAP was measured by immunoblotting. **(P)** An empty vector, Flag-OTUB2 and Flag-OTUB2 C51S mutants were transfected into VSMCs. The K48-linked ubiquitination of YAP was measured by immunoblotting. Statistical significance was assessed using one-way ANOVA followed by Dunnett's test **(E, F)**. All values are presented as mean ± SD. *P < 0.05.

Since OTUB2 modulates YAP activity and VC progression, we next sought to investigate the potential regulatory mechanisms between OTUB2 and YAP. OTUB2, a deubiquitinase, functions mainly by binding to substrate proteins. HDOCK was first performed to evaluate the binding strength between the OTUB2 and YAP proteins (Figure 5A). We then confirmed that OTUB2 interacted with YAP through endogenous Co-IP assays, and the exogenous interaction between OTUB2 and YAP was further confirmed in HEK293T cells transfected with Flag-tagged OTUB2 and HA-tagged YAP (Figure 5B). In parallel, we separated the nuclear and cytoplasmic proteins from the VSMCs and verified that the OTUB2/YAP interaction was detectable in both the cytoplasmic and the nuclear compartments (Figure 5B and Figure S6A). As shown by the immuno-fluorescence results, OTUB2 and YAP were colocalized in VSMCs (Figure 5C). Previous reports have suggested that interactions between OTUB2 and YAP require SUMO modification 36. Our results revealed that SUMOylated OTUB2 levels were apparently increased in VSMCs in response to a procalcific stimulus (Figure 5D). Moreover, Myc-SUMO3 transfection resulted in OTUB2 poly-SUMOylation, which was significantly decreased by the SUMO-specific protease SENP1 (Figure S6B).

We utilized the proteasome inhibitor MG132 and the lysosome inhibitor leupeptin to further clarify how OTUB2 maintains YAP protein stability in VSMCs. OTUB2 silencing decreased the YAP protein level, whereas MG132, but not leupeptin, abrogated this decrease (Figure 5E-F). These results indicated that the maintenance of YAP stability by OTUB2 was mediated by proteasomes but not lysosomes. A protein half-life assay revealed that OTUB2 knockdown decreased YAP protein stability in VSMCs (Figure 5G). Conversely, OTUB2 overexpression increased the stability of YAP (Figure 5H). We generated deletion constructs for an interaction analysis to further elucidate the interaction domains between OTUB2 and YAP. OTUB2 consists of an N-terminal region and a C-terminal OTU domain, whereas the YAP protein is composed of TEAD binding domain (TBD), WW domain, and trans-activation (TA) domain (Figure 5J) 36-37. The results revealed that the OTU domain was necessary for the interaction of OTUB2 with YAP, whereas the TBD domain was required for the interaction of YAP with OTUB2 (Figure 5K-L). Since OTUB2 belongs to the DUB family, we further investigated the effect of OTUB2 on YAP ubiquitination. Ubiquitination assays revealed that OTUB2 silencing significantly increased YAP K48-linked ubiquitination but had little effect on K63-linked ubiquitination, and OTUB2 overexpression significantly decreased YAP K48-linked ubiquitination (Figure 5M-O). Previous studies have shown that the 51st amino acid of the OTUB2 catalytic center is a cysteine molecule, and we changed amino acid 51 from cysteine to serine (C51S) and transfected this gene mutant into VSMCs 37. A protein half-life assay confirmed that OTUB2 C51S could not stabilize the YAP protein (Figure 5I). Additionally, ubiquitination assays revealed that cysteine 51 was critical for the ability of OTUB2 to deubiquitinate YAP (Figure 5P). Taken together, these data suggest that OTUB2 deubiquitylates YAP and promotes its stability in VSMCs.

### OTUB2 facilitates PFKFB3 transcription through YAP

**Figure 6:**
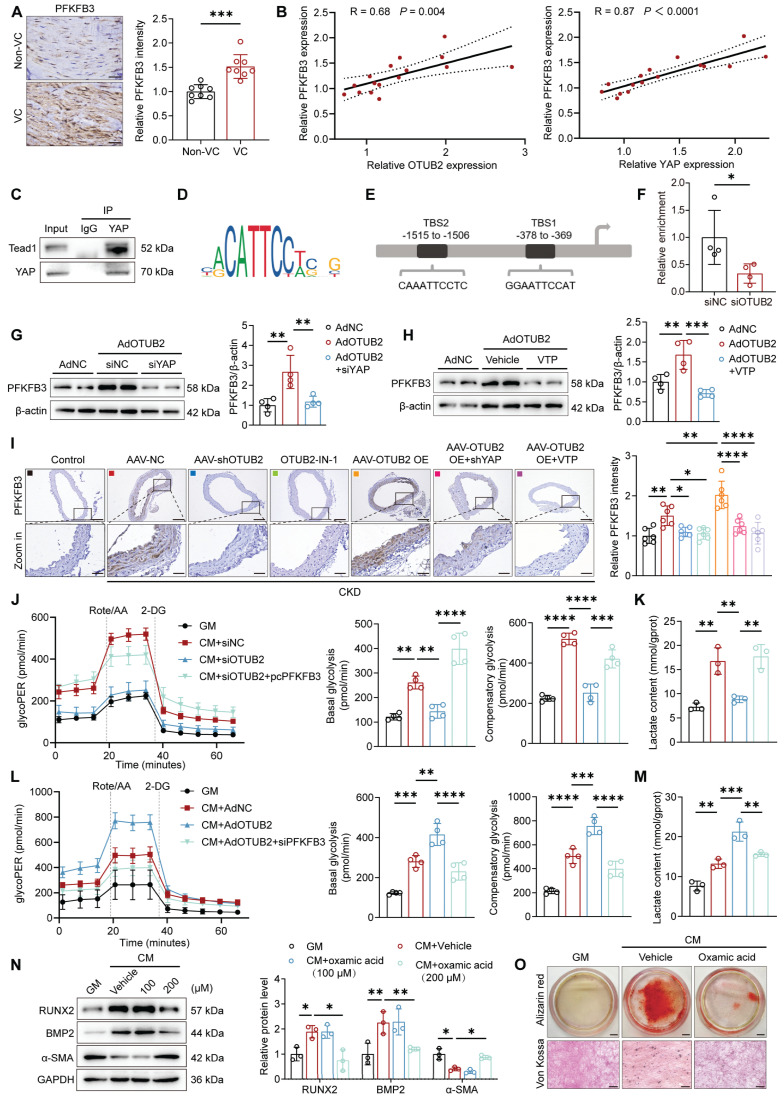
** OTUB2 facilitates PFKFB3 transcription through YAP. (A)** Representative immunohistochemical staining for PFKFB3 of radial arteries from CKD patients. Scale bars, 50 μm. n = 8 per group. **(B)** Scatter dot plot of the correlation between PFKFB3, OTUB2 and YAP expression in radial arteries from CKD patients. n = 16. **(C)** Co-IP assays were performed to verify the interaction between YAP and TEAD1 in VSMCs. **(D)** Binding motif of TEAD1. **(E)** Predicted binding sites of TEAD1 in the promoter of PFKFB3. **(F)** CUT&RUN-qPCR assays were performed to confirm the relative enrichment of genes in VSMCs with the IgG and YAP antibodies. n = 4 per group. (**G-H**) Western blot analysis and quantification of PFKFB3 protein expression in VSMCs after the indicated treatments. n = 4 per group. **(I)** Immunohistochemical staining for PFKFB3 in aortic sections from the indicated groups. Scale bars, 200 μm (upper panels), 50 μm (lower panels). n = 6 per group. **(J)** Glycolysis rate assays were conducted using VSMCs subjected to different treatments with a Seahorse analyzer. n = 4 per group. **(K)** The lactate content was detected after different treatments. n = 3 per group. **(L)** Glycolysis rate assays were conducted using VSMCs subjected to different treatments with a Seahorse analyzer. n = 4 per group. **(M)** The lactate content was detected after different treatments. n = 3 per group. **(N)** Western blot analysis and quantification of RUNX2, BMP2, and α-SMA protein expression in VSMCs after oxamic acid treatment. n = 3 per group. **(O)** Representative images of Alizarin red and Von Kossa staining of VSMCs after the indicated treatments and CM exposure for another 7 days. Scale bars, 5 mm (upper panels), 100 μm (lower panels). Statistical significance was assessed using t-test (A, F) and one-way ANOVA followed by Dunnett's test **(G, H, I, J, K, L, M, N)**. All values are presented as mean ± SD. *P < 0.05, **P < 0.01, ***P < 0.001, and ****P < 0.0001.

Given the crucial role of YAP in the regulation of glycolytic metabolism, we next examined the changes in glycolytic enzyme expression after OTUB2 overexpression. The RT-qPCR analysis demonstrated that OTUB2 overexpression resulted in increased transcript levels of glycolytic enzymes, such as GLUT1, HK2, and PFKFB3 (Figure S7A). Given that PFKFB3 has been reported to be associated with VC, we focused on PFKFB3 as the main downstream target gene of YAP 27-28. Immunohistochemical staining of PFKFB3 was first performed in human radial arteries, consisting of both noncalcified and calcified samples. The results indicated that PFKFB3 expression was significantly increased in tissues from CKD patients with VC (Figure 6A). Notably, increased PFKFB3 expression was positively correlated with upregulated OTUB2 and YAP levels (Figure 6B). Consistently, the elevated PFKFB3 expression in the aortas of CKD model mice was further validated in the GSE159832 dataset (Figure S7B).

As a transcriptional cofactor, YAP is known to bind to transcription factors to exert certain functions, and the TEA domain-containing (TEAD) family is the best characterized 38. TEAD1, a highly conserved protein, was previously reported to promote VSMC proliferation via the induction of solute carrier family 1 member 5 (Slc1a5) transcription 39. We found TEAD1 binding sites within the promoter regions of both human and murine PFKFB3 using the JASPAR database. Co-IP assays were conducted to verify the interaction of YAP with TEAD1 in VSMCs (Figure 6C). Moreover, we performed CUT&RUN-qPCR assays after OTUB2 knockdown to determine whether OTUB2 promotes the binding of the YAP/TEAD1 complex to the PFKFB3 promoter. The results revealed significantly weakened binding of YAP/TEAD1 to the P2 site relative to the TSS in the proximal promoter region of PFKFB3 after the transfection of siOTUB2 (Figure 6D-F and Figure S7C). We next performed Western blot and RT-qPCR experiments to further verify whether the expression of PFKFB3 was regulated by OTUB2 and observed significantly upregulated expression of the PFKFB3 mRNA and protein by OTUB2 overexpression and downregulation after siYAP treatment (Figure 6G and Figure S7D). This result was also confirmed in experiments using VTP, an inhibitor of YAP (Figure 6H and Figure S7E). Consistent with the findings in the VSMC model, the expression levels of PFKFB3 were significantly increased in mouse aortas after OTUB2 overexpression, which was blocked by AAV-shYAP and VTP (Figure 6I).

PFKFB3, a member of the PFKFB family and a crucial rate-limiting glycolytic enzyme, had the highest kinase/phosphatase ratio. This enzyme promotes glycolysis and increases glycolytic flux by accelerating fructose 2,6 bisphosphate (F2,6BP) synthesis 40. Glucose metabolism is a key mechanism associated with the development of VC 11, 26. However, the role of OTUB2 in regulating glycolysis in VSMCs remains largely unknown. We assessed the extracellular acidification rate (ECAR) of VSMCs using an XFe96 extracellular flux analyzer to further explore whether OTUB2 mediates glycolysis through PFKFB3. The results indicated that OTUB2 knockdown decreased the glycolytic proton efflux rate and reduced the basal and compensatory glycolysis levels in VSMCs and that PFKFB3 overexpression significantly abrogated the inhibitory effects of OTUB2 silencing on glycolysis (Figure 6J). Conversely, OTUB2 overexpression further amplified glycolysis in calcified VSMCs, and the stimulatory effects were abrogated by PFKFB3 silencing (Figure 6L). The production of lactate, a key metabolite of glycolysis, was also measured to further validate these findings. As shown in Figure 6K, the increase in the lactate content was decreased in calcified VSMCs treated with siOTUB2, and this phenomenon was abrogated by PFKFB3 overexpression. The reverse experiment further confirmed the above experimental results (Figure 6M). Furthermore, the addition of lactate at the cellular level facilitated VSMC osteogenic differentiation and mineralization upon CM stimulation, consistent with the findings of a previous study (Figure S7F-G) 41. Moreover, oxamic acid sodium, a lactate dehydrogenase inhibitor that decreases lactate production, alleviated VSMC calcification (Figure 6N-O). Collectively, these data indicated that OTUB2 promotes glycolysis in VSMCs by regulating PFKFB3 expression.

### OTUB2 exerts procalcific effects through PFKFB3 upregulation

**Figure 7:**
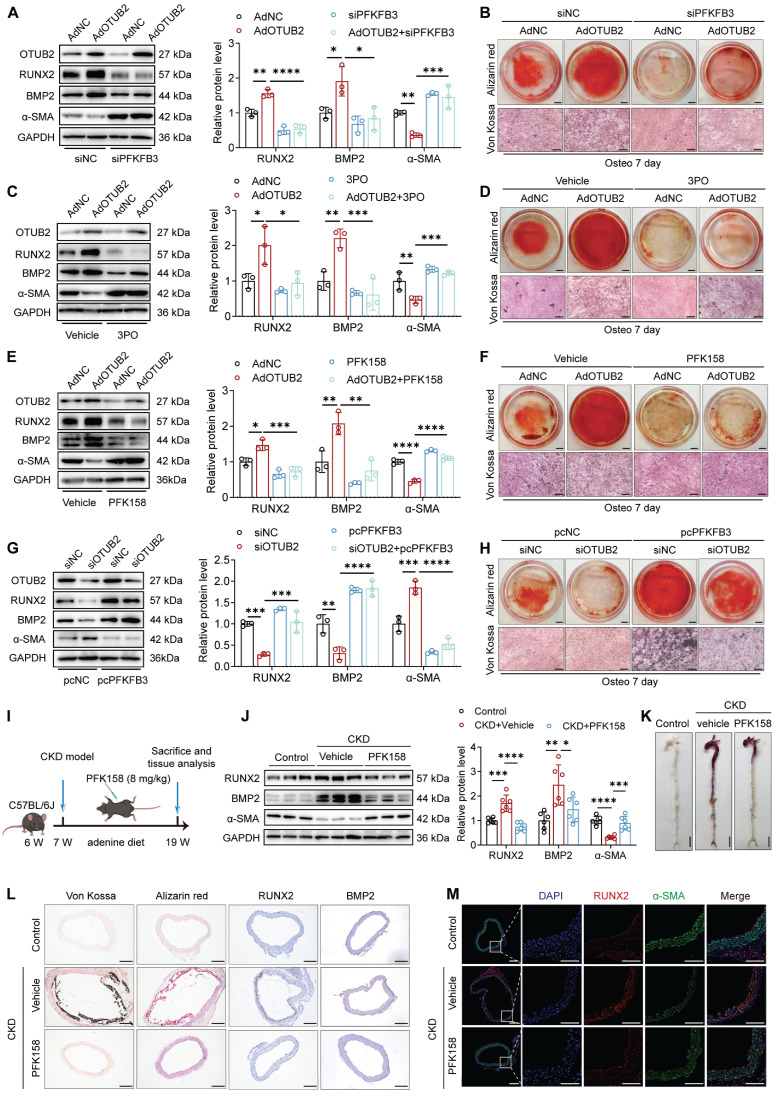
** OTUB2 exerts procalcific effects through PFKFB3 upregulation. (A)** Western blot analysis and quantification of RUNX2, BMP2, and α-SMA protein expression. n = 3 per group. **(B)** Representative images of Alizarin red and Von Kossa staining images. Scale bars, 5 mm (upper panels), 100 μm (lower panels). **(C)** Western blot analysis and quantification of RUNX2, BMP2, and α-SMA protein expression. n = 3 per group. **(D)** Representative images of Alizarin red and Von Kossa staining images. Scale bars, 5 mm (upper panels), 100 μm (lower panels). **(E)** Western blot analysis and quantification of RUNX2, BMP2, and α-SMA protein expression. n = 3 per group. **(F)** Representative images of Alizarin red and Von Kossa staining images. Scale bars, 5 mm (upper panels), 100 μm (lower panels). **(G)** Western blot analysis and quantification of RUNX2, BMP2, and α-SMA protein expression. n = 3 per group. **(H)** Representative images of Alizarin red and Von Kossa staining images. Scale bars, 5 mm (upper panels), 100 μm (lower panels). **(I)** Experimental protocols used in **(J)** through **(M)**. **(J)** Western blot analysis and quantification of RUNX2, BMP2 and α-SMA expression in aortas. n = 6 per group. **(K)** Representative images of Alizarin red staining of whole aortas. Scale bars, 5 mm. **(L)** Representative images of Von Kossa staining, Alizarin red staining, and immunohistochemical staining for RUNX2 and BMP2 in aortas from the indicated experimental cohorts. Scale bars, 200 μm. **(M)** Representative images of immunofluorescence staining for RUNX2 and α-SMA in aortas. Scale bars, 200 μm (left panels), 100 μm (right panels). Statistical significance was assessed using one-way ANOVA followed by Dunnett's test **(A, C, E, G, J)**. All values are presented as mean ± SD. *P < 0.05, **P < 0.01, ***P < 0.001, and ****P < 0.0001.

We utilized a PFKFB3 siRNA to downregulate the expression of PFKFB3 in VSMCs and elucidate whether the upregulation of PFKFB3 is required for the pro-calcification effects of OTUB2 (Figure S8A). The results indicated that the increase in calcium deposition in the VSMCs overexpressing OTUB2 was abrogated in the cells with PFKFB3 deficiency (Figure 7A-B). Additionally, VSMCs transfected with the OTUB2-overexpressing adenovirus were treated with the PFKFB3 inhibitors 3PO and PFK158. We observed that the increased VSMC mineralization induced by OTUB2 overexpression was significantly abrogated following additional treatment with 3PO and PFK158 (Figure 7C-F). Moreover, we showed that PFKFB3 overexpression in VSMCs counteracted the negative regulation of calcium deposition and the anticalcific effect of OTUB2 depletion (Figure 7G-H and Figure S8B).

We performed molecular docking simulations of 3PO or PFK158 with PFKFB3 to identify more effective small-molecule inhibitors of PFKFB3. The results showed that 3PO had a binding energy of -5.8 kcal/mol for PFKFB3, whereas PFK158 had a binding energy of up to -7.4 kcal/mol for PFKFB3 (Figure S8C-D). Finally, to explore whether PFKFB3 is important for promoting VC *in vivo*, we administered PFK158 (8 mg/kg) or the vehicle via intraperitoneal injection to CKD model mice every other day. Figure 7I displays a schematic diagram of the administration of PFK158 therapy to CKD model mice. As expected, after 12 weeks, PFK158 supplementation significantly decreased calcification in aortic tissues (Figure 7J-L, Figure S8E-F and Table S8). The alleviation of VC by PFK158 was further confirmed by dual immunofluorescence staining for RUNX2 and α-SMA (Figure 7M). Together, these findings indicate that the procalcific effects of OTUB2 can be partially suppressed by PFKFB3 knockdown or inhibition, indicating its potential as a novel therapeutic target.

## Discussion

VC contributes significantly to the escalating social and economic burden of CKD 42. Exploring the molecular mechanisms of VC in the context of CKD is highly important. In this study, we revealed that OTUB2 promoted VSMC osteogenic differentiation and aggravated VC both *in vivo* and *in vitro*. Mechanistically, OTUB2 deubiquitylated YAP and promoted its nuclear translocation, leading to increased PFKFB3 expression. The subsequent increase in the PFKFB3 level promoted glycolysis, which resulted in lactate accumulation. This study revealed a novel cross-talk between protein modifications and cellular metabolism during calcification, highlighting OTUB2 as a potential therapeutic target for VC.

VC is an actively regulated process resembling bone morphogenesis 43. OTUB2 is known to regulate the osteogenesis of MSCs by modulating Hedgehog signaling 25. Given the emerging nature of the bone-vascular axis in VC, we first explored changes in OTUB2 expression during the VC process. Although VC can be evaluated using various imaging methods, dynamically assessing VC progression remains challenging. Our observation that OTUB2 expression is markedly upregulated in both mouse and human calcified aortic tissues might propel the development of OTUB2 as a potential biomarker for VC diagnosis and a prognostic factor for CKD patients. Gain- and loss-of-function experiments revealed an important role of OTUB2 in VC. VSMC-specific OTUB2 overexpression caused more severe aortic calcification in CKD mice. Notably, a specific inhibitor developed in a recent study, OTUB2-IN-1, was found to inhibit the catalytic activity of OTUB2 and exert strong inhibitory effects on multiple types of cancer 32. Our data showed that OTUB2-IN-1 treatment suppressed aortic calcification in CKD model mice and VSMC osteogenic transdifferentiation, suggesting that it represents a promising therapeutic strategy for VC. VC is highly prevalent in patients with CKD, and many efforts have been devoted to exploring effective approaches to VC treatment 44. Currently, the approaches that target calcium and phosphate metabolism in treating VC in patients with CKD are still controversial 45. Some novel anti-VC drugs cannot be widely used in the clinic because of their severe side effects 46. Our work provides a new perspective that OTUB2-IN-1, a small-molecule inhibitor that successfully targets OTUB2, may have clinical translational value for VC treatment.

We further explored the mechanism by which OTUB2 promotes calcification by performing RNA-seq to identify changes in gene expression in response to OTUB2 overexpression. The heatmap of genes that were differentially expressed in response to OTUB2 overexpression revealed a group of YAP target genes that were upregulated in VSMCs, which was further confirmed by qPCR experiments. Hyperactivation of YAP expression is linked to a diverse set of cardiovascular diseases, such as cardiac hypertrophy, arteriosclerosis, and myocardial infarction 47-49. A recent study revealed that forced expression of YAP significantly activates RUNX2 reporter gene activity 34. These studies underscore the need to dissect the molecular mechanisms underlying the regulation of YAP protein levels and transcriptional activity. Indeed, YAP protein stability is regulated at multiple levels by complex and interrelated networks, including ubiquitination, methylation, O-GlcNAcylation, and phosphorylation 16-18, 50. Targeting the ubiquitin-proteasome system, which is responsible for YAP protein stability, has broader clinical application prospects. Notably, increasing evidence has demonstrated that the selective inhibition of DUBs may represent an effective approach for the treatment of multiple disorders 51-52.

In the present study, we found that OTUB2 knockdown or inhibition decreased YAP abundance. Neither OTUB2 depletion nor inhibition significantly inhibited YAP nuclear translocation. We also found that knockdown or inhibition of YAP partly abrogated the procalcific effect of OTUB2 on VC models both *in vivo* and *in vitro*. Additionally, inhibition of the proteasome via MG132 prevented the OTUB2-mediated downregulation of the YAP protein. OTUB2 depletion contributed to a shorter half-life of YAP. Furthermore, we identified physical interactions and the interacting domains between the OTUB2 and YAP proteins via Co-IP. As expected, our data revealed that OTUB2 suppressed the polyubiquitination of YAP at the K48 site. Interestingly, we determined that the Cys51 residue is crucial for the enzymatic activity of OTUB2, which coincides with the findings of a previous study indicating that OTUB2 C51S fails to deubiquitinate signal transducer and activator of transcription 1 (STAT1) 53. A previous study revealed a crucial role for SUMOylation in the interaction between OTUB2 and YAP 32. In the present study, we discovered that endogenous OTUB2 SUMOylation is promoted by CM, revealing a potential mechanism of YAP activation under calcifying conditions. However, in the setting of CKD, the specific SUMOylation site of OTUB2 needs to be explored using different methods, such as mass spectrometry. Overall, we showed that OTUB2 is a critical regulator of the ubiquitin-dependent proteolysis of YAP. Additionally, previous studies have shown that the biological function of OTUB2 is complex, and it can activate multiple signaling pathways, such as the NF-κB signaling, AKT/mTOR signaling and Wnt/β-catenin signaling pathways 54-56. These findings were partly consistent with the results of our KEGG enrichment analyses. Whether the aforementioned signaling pathways affect VC development facilitated by OTUB2 was not investigated in this study and warrants further research.

Changes in glucose metabolism is an core contributor to the pathogenesis of VC, and YAP is a key hub of glycolytic metabolism 26, 57. OTUB2-induced changes in metabolic reprogram-ming may be responsible for the accelerated development of VC in CKD. A previous study showed that OTUB2 negatively regulates PKM2 ubiquitination in colorectal cancer 58. Moreover, OTUB2 stimulates the Warburg effect and promotes the progression of non-small cell lung cancer (NSCLC) 55. These studies indicate that OTUB2 may directly or indirectly regulate glucose metabolism through multiple glycolytic enzymes. Interestingly, our RT-qPCR results confirmed that OTUB2 overexpression increased the transcript levels of various glycolytic enzymes in VSMCs, including GLUT1, HK2, PKM2, and PFKFB3. Our study focused mainly on PFKFB3, a key rate-limiting enzyme of glycolysis metabolism. CUT&RUN-qPCR assays showed that OTUB2 silencing suppressed the YAP/TEAD1-promoted transcription of PFKFB3. We further investigated the combined regulatory effects of OTUB2 and PFKFB3 on glycolysis. Analyses using a Seahorse XF96 analyzer revealed that OTUB2 deficiency suppressed glycolytic flux and decreased the production of lactate. These metabolic changes were abolished by PFKFB3 overexpression. Our findings are consistent with those of previous studies in which OTUB2 was implicated as a key regulator of cellular metabolism by altering the expression of multiple metabolic enzymes involved in glycolysis 58-59. Our results suggest that OTUB2 is a novel regulator that targets a protein modification-metabolomic signaling cascade involved in the development of VC. Moreover, the knockdown or inhibition of PFKFB3 counteracted the osteogenic transformation and calcium deposition of VSMCs caused by OTUB2 overactivation. Consistently, we showed that PFKFB3 inhibition by PFK158 alleviated the severity of VC *in vivo*. PFK158 has great potential for hampering the development of atherosclerosis in preclinical models 60. The results of the present study indicated that the mechanism by which PFKFB3 promotes VC development involves increased lactate production 28. Additionally, PFKFB3 is also closely linked to inflammation, an important factor in the development of VC, necessitating further investi-gation of its precise mechanisms 61. Therefore, our findings suggest that the PFKFB3 inhibitor PFK158 may be an effective therapeutic agent for VC.

However, our study has several limitations. First, we should investigate the role of OTUB2 in VC using transgenic VSMC-specific OTUB2 knockout and overexpressing mice to obtain more accurate results. Second, the CKD mouse model induced by an adenine diet exhibited nephrotoxicity and uremia similar to patients with CKD but had the disadvantages of myocardial toxicity and death from dystrophy. In comparison, subtotal nephrectomy surgery (5/6Nx) combined with a high-phosphorus diet in mice simulated the progressive renal dysfunction and nephron decline observed in humans with chronic renal failure (CRF) but was associated with relatively high mortality. A variety of CKD models must be applied to better clarify the pathogenesis of CKD-related VC. Additionally, a VSMC calcification model induced by inorganic phosphorus should be utilized to further confirm our conclusions. Third, although we found that OTUB2 promotes the transcription of key glycolytic enzymes, including GLUT1, HK2, and PKM2, we cannot exclude the possibility that OTUB2 may regulate other glycolytic enzymes in addition to PFKFB3 to affect VC development. Fourth, although we determined that OTUB2 promoted glycolysis and increased the lactate content in VSMCs, we did not investigate whether OTUB2 regulates histone lactylation levels during VC progression.

## Conclusions

In summary, we observed that OTUB2 is a pivotal regulator of YAP in calcified VSMCs, increasing PFKFB3 expression and contributing to the development of VC. This study provides a new perspective on the connection between protein modifications and metabolic reprogramming. Thus, targeting OTUB2 could be a promising therapeutic strategy for VC.

## Supplementary Material

Supplementary figures and tables.

## Abbreviations

AAV: adeno-associated virus
ALP: alkaline phosphatase
α-SMA: alpha-smooth muscle actin
BMP2: bone morphogenetic protein 2
CKD: chronic kidney disease
CM: calcifying media
DEGs: differentially expressed genes
DUBs: deubiquitinases
ESRD: end-stage renal disease
GLUT1: glucose transporter 1
GM: growth medium
HEK293: human embryonic kidney 293
HK2: hexokinase 2
IHC: immunohistochemistry
IF: immunofluorescence
KEGG: kyoto encyclopedia of genes and genomes
MSCs: mesenchymal stem cells
MDCT: multi-detector computed tomography
NSCLC: non-small cell lung cancer
OTUB1: otubain-1
OTUB2: otubain-2
PFKFB3: 6-phosphofructo-2-kinase/fructose-2,6-bisphosphatase isoform 3
PKM2: pyruvate kinase M2
RUNX2: runt-related transcription factor 2
siRNA: small interfering RNA
Slc1a5: solute carrier family 1 member 5
STAT1: signal transducer and activator of transcription 1
TEAD: TEA domain-containing
Ub: ubiquitin
VC: vascular calcification
VSMCs: vascular smooth muscle cells
VTP: verteporfin
YAP: yes-associated protein

## References

[1] Onnis C, Virmani R, Kawai K, Nardi V, Lerman A, Cademartiri F (2024). Coronary artery calcification: current concepts and clinical implications. Circulation.

[2] Dong QQ, Tu YC, Gao P, Liao QQ, Zhou P, Zhang H (2024). SGK3 promotes vascular calcification via Pit-1 in chronic kidney disease. Theranostics.

[3] Zhang H, Li G, Yu X, Yang J, Jiang A, Cheng H (2023). Progression of vascular calcification and clinical outcomes in patients receiving maintenance dialysis. JAMA Netw Open.

[4] Li X, Liu A, Xie C, Chen Y, Zeng K, Xie C (2024). The transcription factor GATA6 accelerates vascular smooth muscle cell senescence-related arterial calcification by counteracting the role of anti-aging factor SIRT6 and impeding DNA damage repair. Kidney Int.

[5] Sutton NR, Malhotra R, St Hilaire C, Aikawa E, Blumenthal RS, Gackenbach G (2023). Molecular mechanisms of vascular health: insights from vascular aging and calcification. Arterioscler Thromb Vasc Biol.

[6] Li Q, Zhang C, Shi J, Yang Y, Xing X, Wang Y (2022). High-phosphate-stimulated macrophage-derived exosomes promote vascular calcification via let-7b-5p/TGFBR1 axis in chronic kidney disease. Cells.

[7] Lin X, Shan SK, Xu F, Zhong JY, Wu F, Duan JY (2022). The crosstalk between endothelial cells and vascular smooth muscle cells aggravates high phosphorus-induced arterial calcification. Cell Death Dis.

[8] Liu A, Chen Z, Li X, Xie C, Chen Y, Su X (2023). C5a-C5aR1 induces endoplasmic reticulum stress to accelerate vascular calcification via PERK-eIF2α-ATF4-CREB3L1 pathway. Cardiovasc Res.

[9] Ortega MA, De Leon-Oliva D, Gimeno-Longas MJ, Boaru DL, Fraile-Martinez O, García-Montero C (2024). Vascular calcification: molecular networking, pathological implications and translational opportunities. Biomolecules.

[10] Zhang X, Zhao Q, Wang T, Long Q, Sun Y, Jiao L (2023). DNA damage response, a double-edged sword for vascular aging. Ageing Res Rev.

[11] Rashdan NA, Sim AM, Cui L, Phadwal K, Roberts FL, Carter R (2020). Osteocalcin regulates arterial calcification via altered Wnt signaling and glucose metabolism. J Bone Miner Res.

[12] Pan C, Hao X, Deng X, Lu F, Liu J, Hou W (2024). The roles of Hippo/YAP signaling pathway in physical therapy. Cell Death Discov.

[13] Shi Y, Jin X, Yang M, Jia J, Yao H, Yuan W (2022). CKAP4 contributes to the progression of vascular calcification (VC) in chronic kidney disease (CKD) by modulating YAP phosphorylation and MMP2 expression. Cell Signal.

[14] Yang C, Xie Z, Liu H, Wang X, Zhang Z, Du L (2023). Efficacy and mechanism of Shenqi Compound in inhibiting diabetic vascular calcification. Mol Med.

[15] Liu J, Wang J, Liu Y, Xie SA, Zhang J, Zhao C (2023). Liquid-liquid phase separation of DDR1 counteracts the Hippo pathway to orchestrate arterial stiffening. Circ Res.

[16] Qi J, Zhang X, Zhang S, Wu S, Lu Y, Li S (2023). P65 mediated UBR4 in exosomes derived from menstrual blood stromal cells to reduce endometrial fibrosis by regulating YAP Ubiquitination. J Nanobiotechnology.

[17] Namoto K, Baader C, Orsini V, Landshammer A, Breuer E, Dinh KT (2024). NIBR-LTSi is a selective LATS kinase inhibitor activating YAP signaling and expanding tissue stem cells *in vitro* and in vivo. Cell Stem Cell.

[18] Xu Y, Song M, Hong Z, Chen W, Zhang Q, Zhou J (2023). The N6-methyladenosine METTL3 regulates tumorigenesis and glycolysis by mediating m6A methylation of the tumor suppressor LATS1 in breast cancer. J Exp Clin Cancer Res.

[19] Yang L, Dai R, Wu H, Cai Z, Xie N, Zhang X (2022). Unspliced XBP1 counteracts β-Catenin to inhibit vascular calcification. Circ Res.

[20] Li W, Feng W, Su X, Luo D, Li Z, Zhou Y (2022). SIRT6 protects vascular smooth muscle cells from osteogenic transdifferentiation via Runx2 in chronic kidney disease. J Clin Invest.

[21] Zhou Y, Chu P, Wang Y, Li N, Gao Q, Wang S (2024). Epinephrine promotes breast cancer metastasis through a ubiquitin-specific peptidase 22-mediated lipolysis circuit. Sci Adv.

[22] Kassel S, Hanson AJ, Benchabane H, Saito-Diaz K, Cabel CR, Goldsmith L (2023). USP47 deubiquitylates Groucho/TLE to promote Wnt-β-catenin signaling. Sci Signal.

[23] Kato K, Nakajima K, Ui A, Muto-Terao Y, Ogiwara H, Nakada S (2014). Fine-tuning of DNA damage-dependent ubiquitination by OTUB2 supports the DNA repair pathway choice. Mol Cell.

[24] Zhu Q, Fu Y, Li L, Liu CH, Zhang L (2021). The functions and regulation of Otubains in protein homeostasis and diseases. Ageing Res Rev.

[25] Li XY, Mao XF, Tang XQ, Han QQ, Jiang LX, Qiu YM (2018). Regulation of Gli2 stability by deubiquitinase OTUB2. Biochem Biophys Res Commun.

[26] Ma W, Jia K, Cheng H, Xu H, Li Z, Zhang H (2024). Orphan nuclear receptor NR4A3 promotes vascular calcification via histone lactylation. Circ Res.

[27] Niu J, Wu C, Zhang M, Yang Z, Liu Z, Fu F (2021). κ-opioid receptor stimulation alleviates rat vascular smooth muscle cell calcification via PFKFB3-lactate signaling. Aging (Albany NY).

[28] Chen J, Yu H, Tan X, Mok SWF, Xie Y, Wang Y (2023). PFKFB3-driven vascular smooth muscle cell glycolysis promotes vascular calcification via the altered FoxO3 and lactate production. FASEB J.

[29] Li Y, Chen X, Xiong Y, Xu X, Xie C, Min M (2024). BRCC36 regulates β-catenin ubiquitination to alleviate vascular calcification in chronic kidney disease. J Transl Med.

[30] Zhu Q, Fu Y, Cui CP, Ding Y, Deng Z, Ning C (2023). OTUB1 promotes osteoblastic bone formation through stabilizing FGFR2. Signal Transduct Target Ther.

[31] Xu F, Chen H, Zhou C, Zang T, Wang R, Shen S (2024). Targeting deubiquitinase OTUB1 protects vascular smooth muscle cells in atherosclerosis by modulating PDGFRβ. Front Med.

[32] Ren W, Xu Z, Chang Y, Ju F, Wu H, Liang Z (2024). Pharmaceutical targeting of OTUB2 sensitizes tumors to cytotoxic T cells via degradation of PD-L1. Nat Commun.

[33] Wang L, You X, Lotinun S, Zhang L, Wu N, Zou W (2020). Mechanical sensing protein PIEZO1 regulates bone homeostasis via osteoblast-osteoclast crosstalk. Nat Commun.

[34] McNeill MC, Li Mow Chee F, Ebrahimighaei R, Sala-Newby GB, Newby AC, Hathway T (2024). Substrate stiffness promotes vascular smooth muscle cell calcification by reducing the levels of nuclear actin monomers. J Mol Cell Cardiol.

[35] Zhang F, Sahu V, Peng K, Wang Y, Li T, Bala P (2024). Recurrent RhoGAP gene fusion CLDN18-ARHGAP26 promotes RHOA activation and focal adhesion kinase and YAP-TEAD signalling in diffuse gastric cancer. Gut.

[36] Zhang Z, Du J, Wang S, Shao L, Jin K, Li F (2019). OTUB2 promotes cancer metastasis via Hippo-independent activation of YAP and TAZ. Mol Cell.

[37] Balakirev MY, Tcherniuk SO, Jaquinod M, Chroboczek J (2003). Otubains: a new family of cysteine proteases in the ubiquitin pathway. EMBO Rep.

[38] Ju J, Zhang H, Lin M, Yan Z, An L, Cao Z (2024). The alanyl-tRNA synthetase AARS1 moonlights as a lactyltransferase to promote YAP signaling in gastric cancer. J Clin Invest.

[39] Osman I, He X, Liu J, Dong K, Wen T, Zhang F (2019). TEAD1 (TEA Domain Transcription Factor 1) promotes smooth muscle cell proliferation through upregulating SLC1A5 (Solute Carrier Family 1 Member 5)-mediated glutamine uptake. Circ Res.

[40] Wang Y, Li H, Jiang S, Fu D, Lu X, Lu M (2024). The glycolytic enzyme PFKFB3 drives kidney fibrosis through promoting histone lactylation-mediated NF-κB family activation. Kidney Int.

[41] Zhu Y, Han XQ, Sun XJ, Yang R, Ma WQ, Liu NF (2020). Lactate accelerates vascular calcification through NR4A1-regulated mitochondrial fission and BNIP3-related mitophagy. Apoptosis.

[42] Lu Y, Meng L, Ren R, Wang X, Sui W, Xue F (2024). Paraspeckle protein NONO attenuates vascular calcification by inhibiting bone morphogenetic protein 2 transcription. Kidney Int.

[43] Paloian NJ, Giachelli CM (2014). A current understanding of vascular calcification in CKD. Am J Physiol Renal Physiol.

[44] Palit S, Kendrick J (2014). Vascular calcification in chronic kidney disease: role of disordered mineral metabolism. Curr Pharm Des.

[45] Xu C, Smith ER, Tiong MK, Ruderman I, Toussaint ND (2022). Interventions to attenuate vascular calcification progression in chronic kidney disease: a systematic review of clinical trials. J Am Soc Nephrol.

[46] Chen CL, Chen NC, Wu FZ, Wu MT (2020). Impact of denosumab on cardiovascular calcification in patients with secondary hyperparathyroidism undergoing dialysis: a pilot study. Osteoporos Int.

[47] Kashihara T, Mukai R, Oka SI, Zhai P, Nakada Y, Yang Z (2022). YAP mediates compensatory cardiac hypertrophy through aerobic glycolysis in response to pressure overload. J Clin Invest.

[48] Jia M, Li Q, Guo J, Shi W, Zhu L, Huang Y (2022). Deletion of BACH1 attenuates atherosclerosis by reducing endothelial inflammation. Circ Res.

[49] Huang HC, Wang TY, Rousseau J, Orlando M, Mungaray M, Michaud C (2024). Biomimetic nanodrug targets inflammation and suppresses YAP/TAZ to ameliorate atherosclerosis. Biomaterials.

[50] Zhang X, Qiao Y, Wu Q, Chen Y, Zou S, Liu X (2017). The essential role of YAP O-GlcNAcylation in high-glucose-stimulated liver tumorigenesis. Nat Commun.

[51] Gulla A, Morelli E, Johnstone M, Turi M, Samur MK, Botta C (2024). Loss of GABARAP mediates resistance to immunogenic chemotherapy in multiple myeloma. Blood.

[52] Voorhees PM, Sborov DW, Laubach J, Kaufman JL, Reeves B, Rodriguez C (2023). Addition of daratumumab to lenalidomide, bortezomib, and dexamethasone for transplantation-eligible patients with newly diagnosed multiple myeloma (GRIFFIN): final analysis of an open-label, randomised, phase 2 trial. Lancet Haematol.

[53] Chang W, Luo Q, Wu X, Nan Y, Zhao P, Zhang L (2022). OTUB2 exerts tumor-suppressive roles via STAT1-mediated CALML3 activation and increased phosphatidylserine synthesis. Cell Rep.

[54] Gu ZL, Huang J, Zhen LL (2020). Knockdown of otubain 2 inhibits liver cancer cell growth by suppressing NF-κB signaling. Kaohsiung J Med Sci.

[55] Li J, Cheng D, Zhu M, Yu H, Pan Z, Liu L (2019). OTUB2 stabilizes U2AF2 to promote the warburg effect and tumorigenesis via the AKT/mTOR signaling pathway in non-small cell lung cancer. Theranostics.

[56] Xu X, Wu G, Han K, Cui X, Feng Y, Mei X (2023). Inhibition of OTUB2 suppresses colorectal cancer cell growth by regulating β-Catenin signaling. Am J Cancer Res.

[57] Xie B, Lin J, Chen X, Zhou X, Zhang Y, Fan M (2023). CircXRN2 suppresses tumor progression driven by histone lactylation through activating the Hippo pathway in human bladder cancer. Mol Cancer.

[58] Yu S, Zang W, Qiu Y, Liao L, Zheng X (2022). Deubiquitinase OTUB2 exacerbates the progression of colorectal cancer by promoting PKM2 activity and glycolysis. Oncogene.

[59] Zhang Y, Zhang H, Wang C, Cao S, Cheng X, Jin L (2024). circRNA6448-14/miR-455-3p/OTUB2 axis stimulates glycolysis and stemness of esophageal squamous cell carcinoma. Aging (Albany NY).

[60] Guo S, Li A, Fu X, Li Z, Cao K, Song M (2022). Gene-dosage effect of Pfkfb3 on monocyte/macrophage biology in atherosclerosis. Br J Pharmacol.

[61] Wang X, Liu X, Wu W, Liao L, Zhou M, Wang X (2024). Hypoxia activates macrophage-NLRP3 inflammasome promoting atherosclerosis via PFKFB3-driven glycolysis. FASEB J.

