# Supplementary material for: OTUB2 contributes to vascular calcification in chronic kidney disease via the YAP-mediated transcription of PFKFB3
Source: Theranostics. 2025 Jan 1;15(3):1185–204. doi: 10.7150/thno.98660 (PMC11700865; doi:10.7150/thno.98660)
Supplement: Supplementary file 1 — Supplementary figures and tables. [file thnov15p1185s1.pdf]

Title page

OTUB2 contributes to vascular calcification in chronic kidney disease via the YAP-mediated transcription of PFKFB3

Yalan Li[1], Xiaoyue Chen[1], Xueqiang Xu[1], Cheng Chen[2], Min Min[1], Dongmei Liang[1], Jiafa Ren[1], Huijuan Mao[1]

Author Affiliations: 1. Department of Nephrology, Jiangsu Province Hospital, The First Affiliated Hospital of Nanjing Medical University, Nanjing Medical University, 300 Guangzhou Road, Nanjing 210029, China. 2. Department of Medical Science, Yangzhou Polytechnic College, 458 West Wenchang Road, Yangzhou 225009, China.

Corresponding Authors: Huijuan Mao, MD, PhD, Department of Nephrology, Jiangsu Province Hospital, The First Affiliated Hospital of Nanjing Medical University, Nanjing Medical University, 300 Guangzhou Road, Nanjing 210029, China; Telephone number: +86-13851746819; E-mail: maohuijuan72@njmu.edu.cn; Jiafa Ren, PhD, Department of Nephrology, Jiangsu Province Hospital, The First Affiliated Hospital of Nanjing Medical University, Nanjing Medical University, 300 Guangzhou Road, Nanjing 210029, China; Telephone number: +86-13952012989; E-mail: jfren@njmu.edu.cn.

Category: Original Article

Supplemental figures

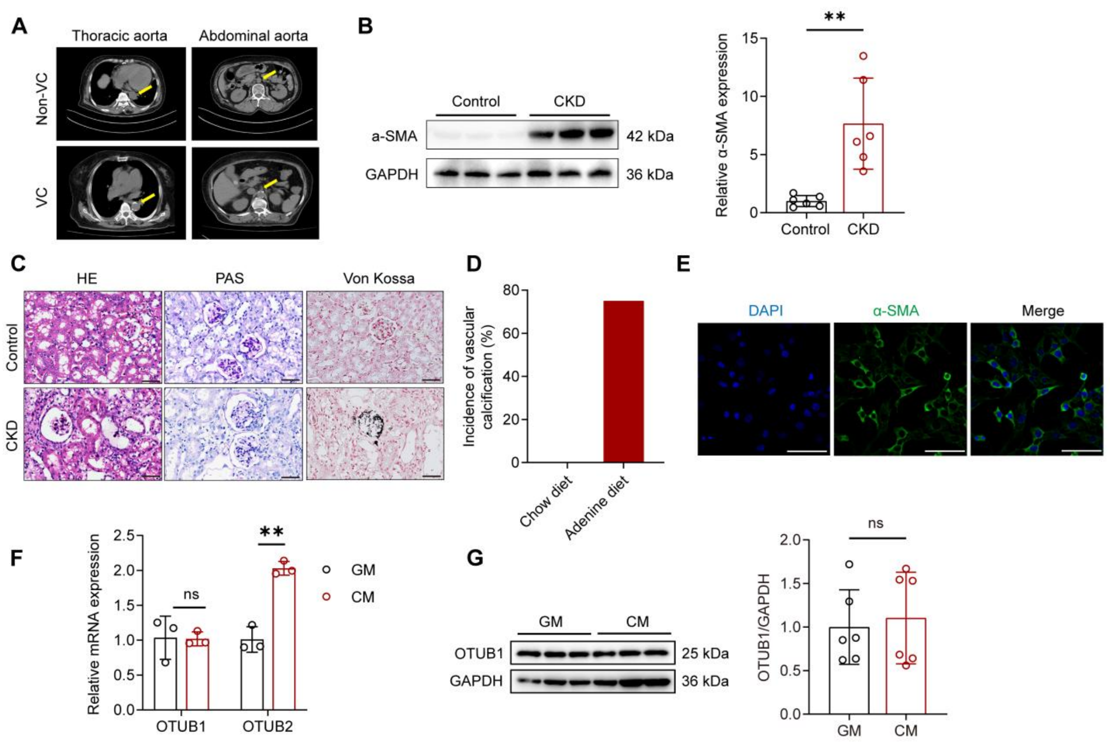

Figure S1. OTUB2 expression is upregulated during VC. (**A**) Representative images of MDCT scans of thoracic aorta and abdominal aorta of CKD patients with or without VC. (**B**) Western blot analysis of α-SMA expression in kidneys from the control and CKD groups. n = 6 per group. (**C**) Representative images of HE, PAS, and Von Kossa staining of kidneys from the control and CKD groups. Scale bars, 50 µm. (**D**) The incidence of vascular calcification in mice fed with a chow or adenine diet. (**E**) The primary cells isolated from mouse aorta were identified by VSMC marker (α-SMA) in immunofluorescence staining. Scale bars, 100 µm. (**F**) RT-qPCR analysis of OTUB1 and OTUB2 mRNA expression in VSMCs treated with GM or CM. n = 3 per group. (**G**) Immunoblots and quantification of OTUB1 in VSMCs treated with GM or CM. n = 6 per group. Statistical significance was assessed using two-tailed t-test (B, F, G). All values are presented as mean ± SD. $^{**}P < 0.01$.

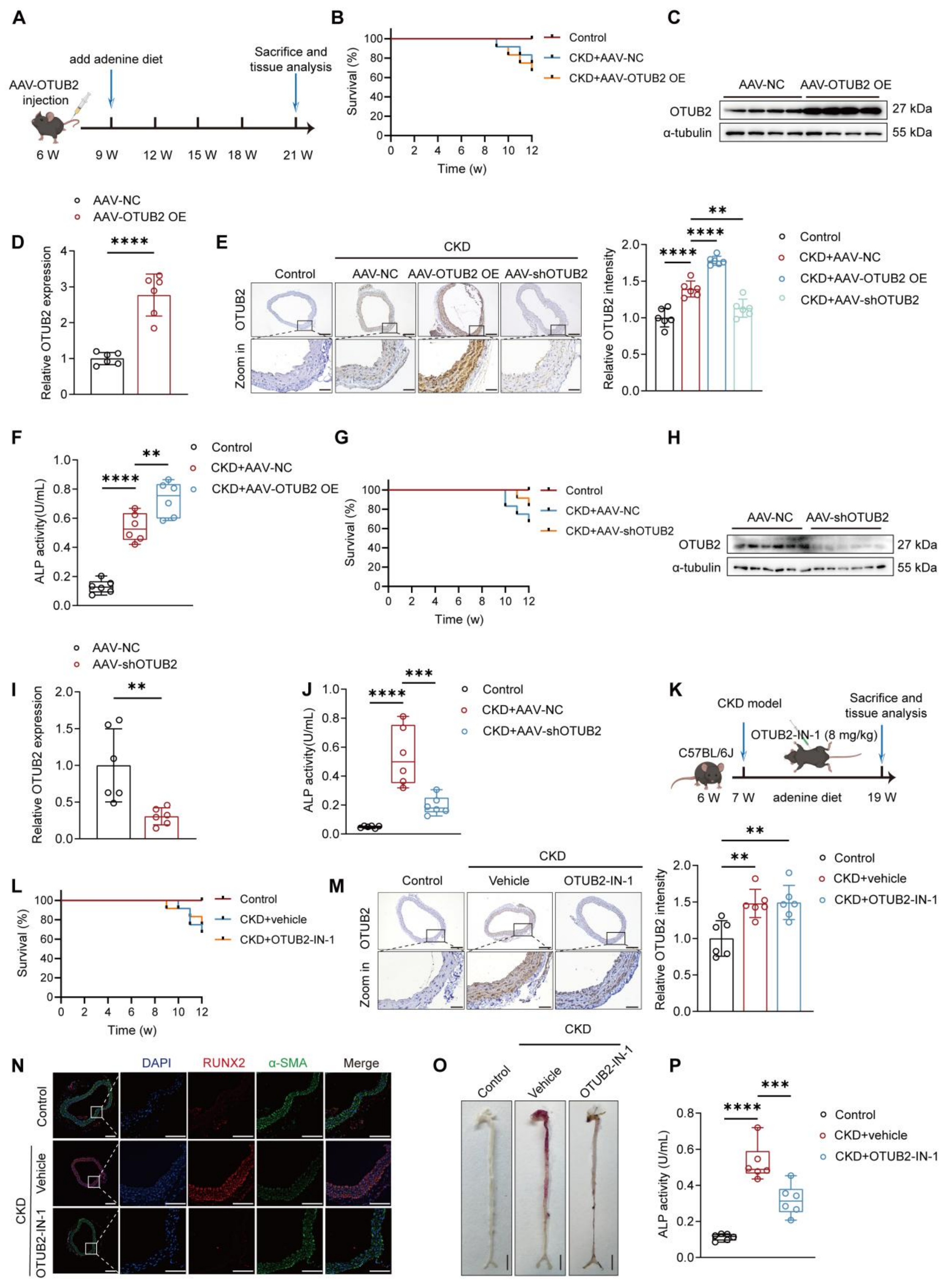

Figure S2. OTUB2 accelerates the development of VC. (**A**) A schematic representation showing that mice received AAV-NC, AAV-OTUB2 OE and AAV-shOTUB2 via tail vein injection. Mouse aortas were collected 12 weeks after adenine diet. (**B**) The survival curves of mice in each group. (**C**) Western blot and quantification (**D**) analysis identifying the OTUB2 overexpression in aortas. n = 6 per group.

(**E**) Representative images of immunohistochemical staining for OTUB2 to validate overexpression and knockdown efficiency. Scale bars, 200 μm (upper panels), 50 μm (lower panels). n = 6 per group. (**F**) ALP activity assay. n = 6 per group. (**G**) The survival curves of mice in each group. (**H**) Western blot and quantification (**I**) analysis identifying the OTUB2 deficiency in aortas. n = 6 per group. (**J**) ALP activity assay. n = 6 per group. (**K**) A schematic representation showing that mice received OTUB2-IN-1 via intraperitoneal injection. (**L**) The survival curves of mice in each group. (**M**) Representative images of immunohistochemical staining for OTUB2. Scale bars, 200 μm (upper panels), 50 μm (lower panels). n = 6 per group. (**N**) Representative images of immunofluorescence staining for RUNX2 and α-SMA in aortas from the different experimental groups. Scale bars, 200 μm (left panels), 100 μm (right panels). (**O**) Representative images of Alizarin red staining of whole aortas from different experimental groups. Scale bars, 5 mm. (**P**) ALP activity assay. n = 6 per group. Statistical significance was assessed using two-tailed t-test (D, I) and one-way ANOVA followed by Dunnett's test (E, F, J, M, P). All values are presented as mean ± SD. **P* < 0.05, ***P* < 0.01, ****P* < 0.001.

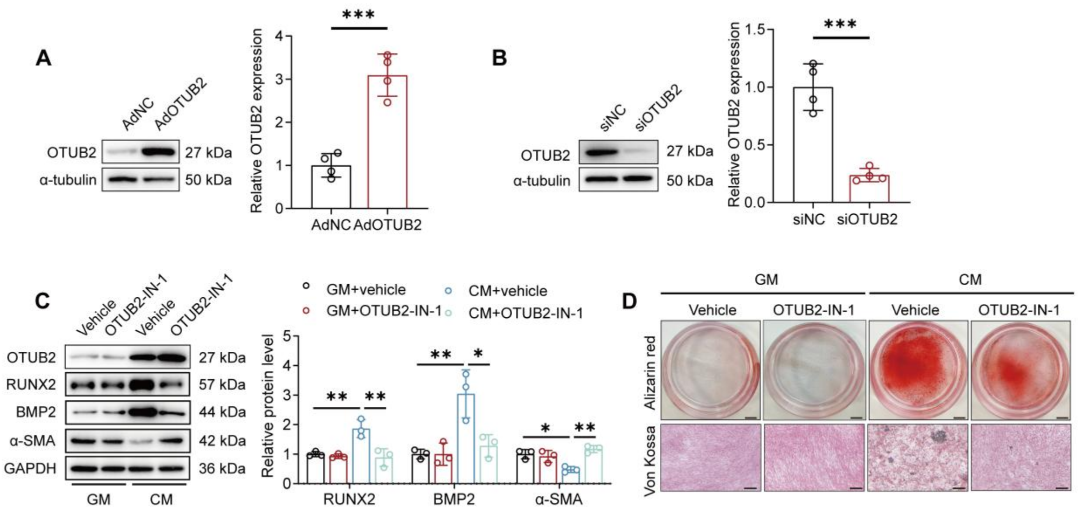

Supplemental Figure S3. OTUB2 regulates osteogenic differentiation of VSMCs. (**A**) Western blot

analysis identifying the OTUB2 overexpression in VSMCs. n = 4 per group. (**B**) Western blot analysis identifying the OTUB2 knockdown in VSMCs. n = 4 per group. (**C**) Western blot analysis of OTUB2, RUNX2, BMP2, and α-SMA expression in VSMCs with OTUB2 inhibition by OTUB2-IN-1. n = 3 per group. (**D**) Representative images of Alizarin red and Von Kossa staining of VSMCs after OTUB2-IN-1 treatment (10 μM) and CM exposure for another 7 days. Scale bars, 5 mm (upper panels), 100 μm (lower panels). Statistical significance was assessed using two-tailed t-test (A, B) and one-way ANOVA followed by Dunnett's test (C). All values are presented as mean ± SD. *$P < 0.05$, **$P < 0.01$, ***$P <$ 0.001.

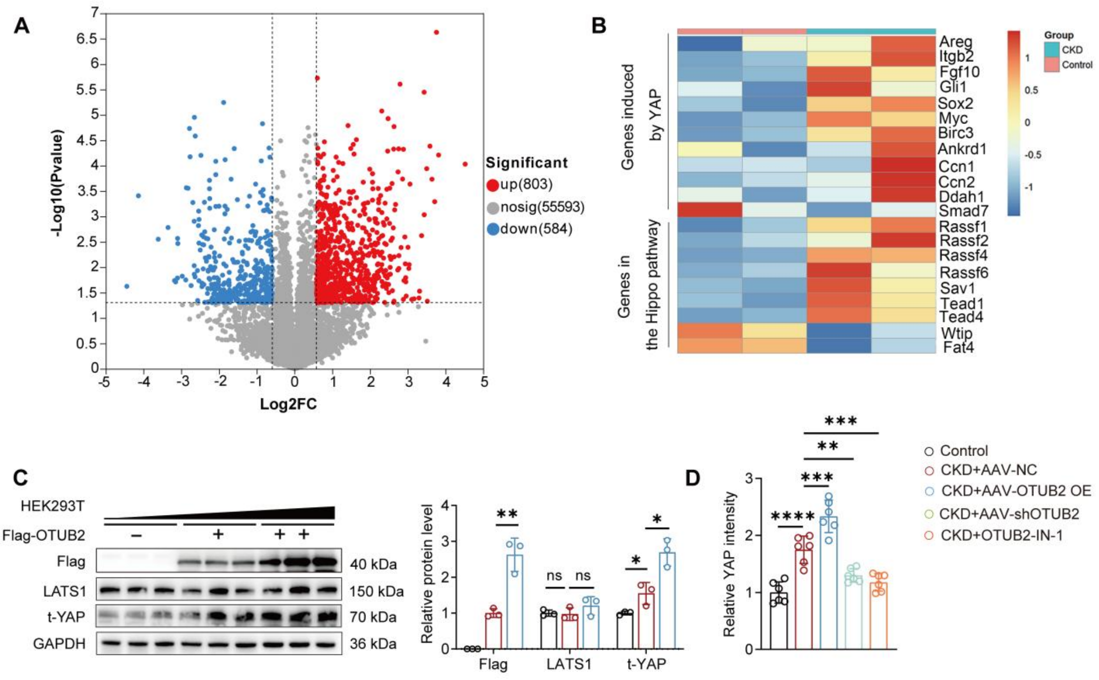

Supplemental Figure S4. OTUB2 activates YAP in VSMCs. (**A**) Volcano map of RNA-seq data from VSMCs treated with control adenoviruses or adenoviruses overexpressing OTUB2. (**B**) Heatmap of differentially expressed YAP targeted genes in RNA-seq data from the GSE159832 dataset. (**C**) Western blot analysis and quantification of YAP and LATS1 levels in HEK293T cells. n = 3 per group. (**D**) Quantification of YAP protein levels in aortic sections from the indicated groups. n = 6 per group. Statistical significance was assessed using one-way ANOVA followed by Dunnett's test (C, D). All

values are presented as mean ± SD. *$P < 0.05$, **$P < 0.01$, ***$P < 0.001$, and ****$P < 0.0001$.

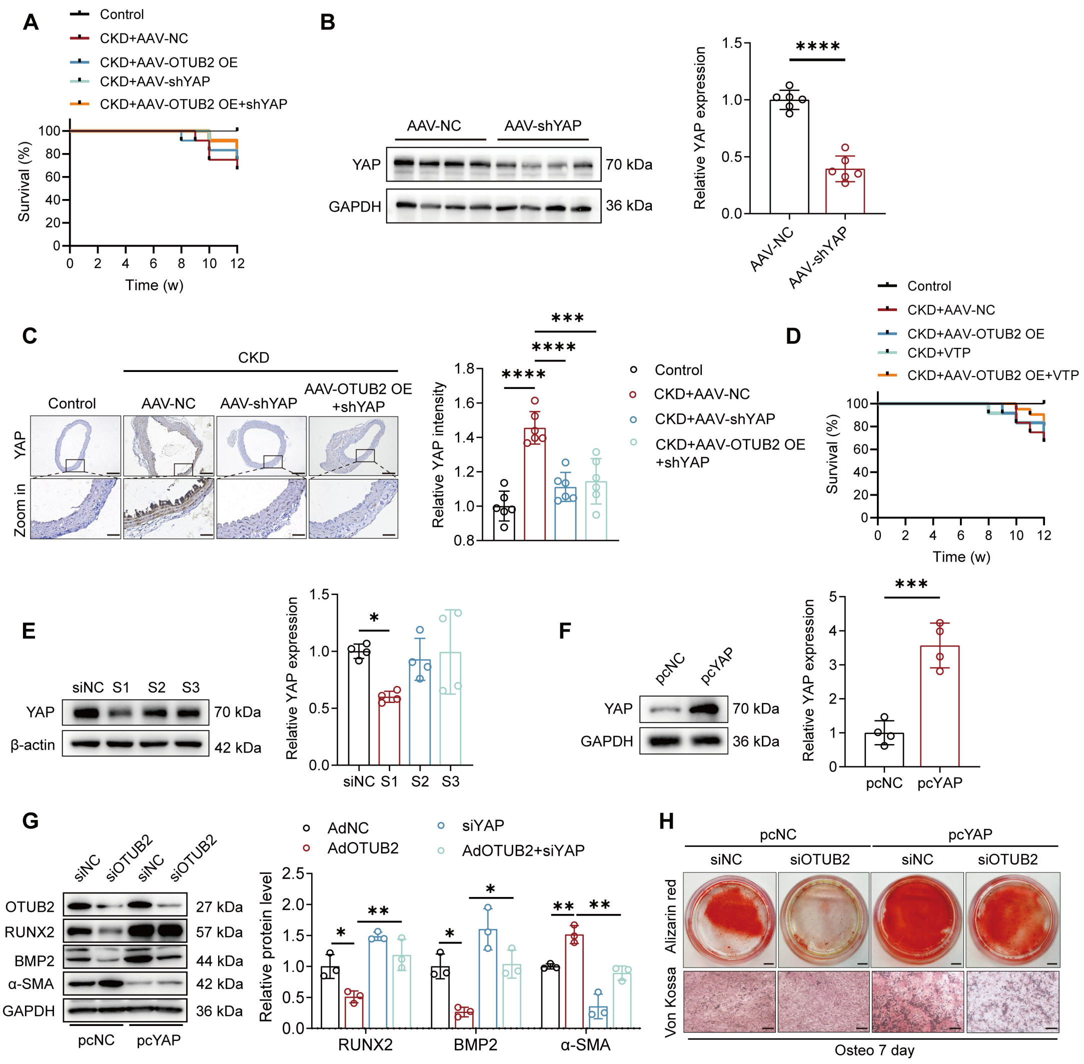

Supplemental Figure S5. Knockdown or inhibition of YAP partially suppresses VC promoted by OTUB2 overexpression. (**A**) The survival curves of mice in each group. (**B**) YAP knockdown efficiency was confirmed by Western blot analysis. n = 6 per group. (**C**) YAP knockdown efficiency was confirmed by immunohistochemical staining. Scale bars, 200 μm (upper panels) and 50 μm (lower panels). n = 6 per group. (**D**) The survival curves of mice in each group. (**E**) Western blot analysis identifying the YAP knockdown in VSMCs. n = 4 per group. (**F**) Western blot analysis identifying the YAP overexpression in VSMCs. n = 4 per group. (**G**) Western blot analysis of OTUB2, RUNX2, BMP2, and α-SMA expression in VSMCs. n = 3 per group. (**H**) Representative images of Alizarin red and Von

Kossa staining of VSMCs after the indicated treatments and CM exposure for another 7 days. Scale bars, 5 mm (upper panels) and 100 μm (lower panels). Statistical significance was assessed using two-tailed t-test (B, F) and one-way ANOVA followed by Dunnett's test (C, E, G). All values are presented as mean ± SD. *$P < 0.05$, **$P < 0.01$, ***$P < 0.001$, and ****$P < 0.0001$.

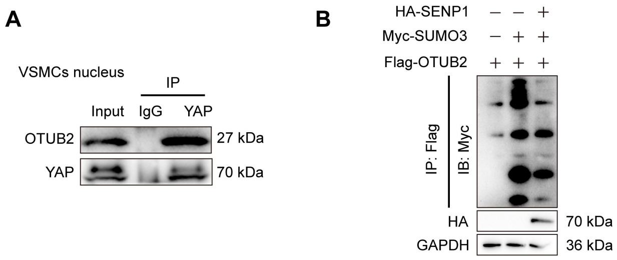

Supplemental Figure S6. OTUB2 interacts with and stabilizes YAP. (**A**) Nuclear lysates of VSMCs were immunoprecipitated using an anti-YAP antibody. (**B**) Western blot analysis of cell lysates and anti-Flag immunoprecipitates of cells co-transfected with Myc-SUMO3, Flag-OTUB2, and HA-SENP1.

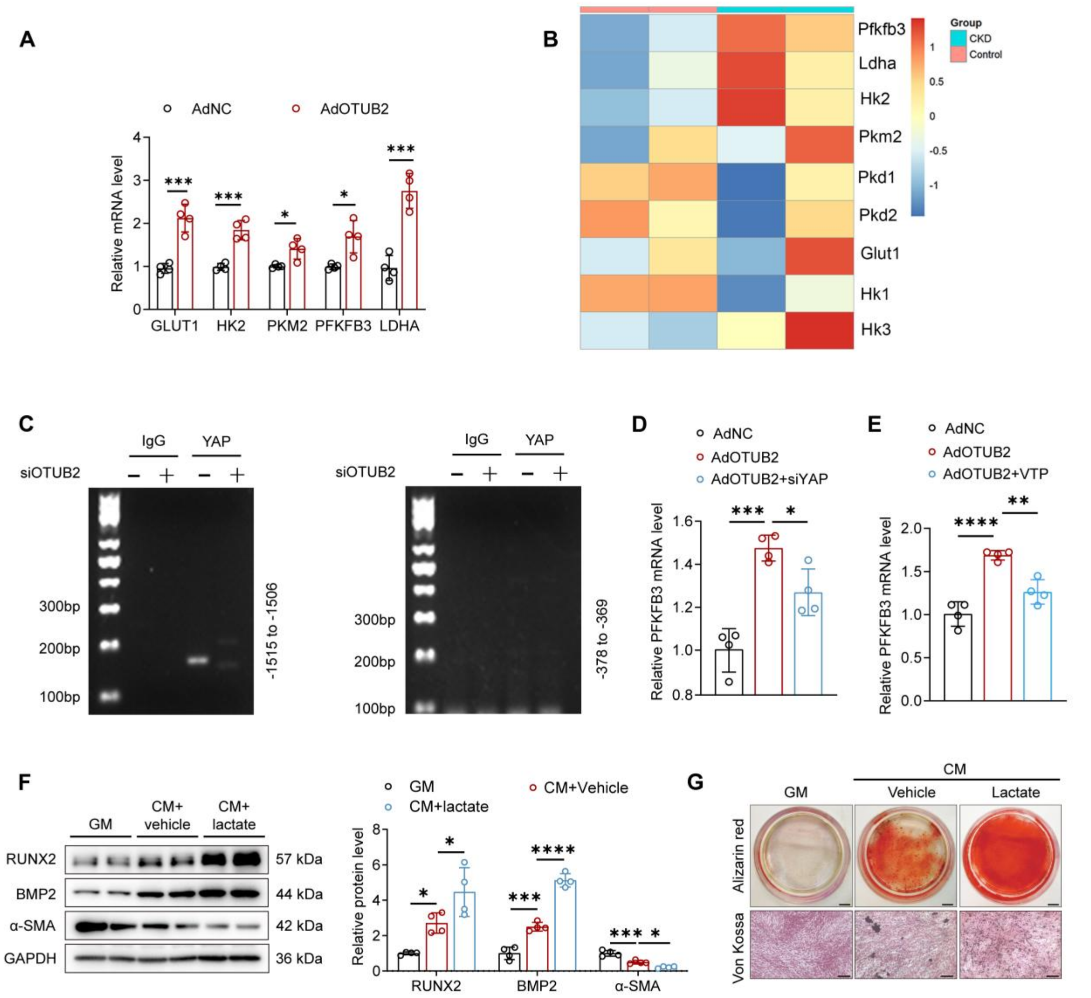

Supplemental Figure S7. OTUB2 facilitates PFKFB3 transcription through YAP. (**A**) RT-qPCR analysis of glycolysis-related enzymes mRNA levels after OTUB2 overexpression. n = 4 per group. (**B**) Heatmap of differentially expressed glycolytic enzymes in RNA-seq data from the GSE159832 dataset. (**C**) Representative agarose gel results for CUT&RUN-qPCR assays. (**D-E**) RT-qPCR analysis of PFKFB3 mRNA levels after the indicated treatments. n = 4 per group. (**F**) Western blot analysis and quantification of RUNX2, BMP2, and α-SMA protein expression in VSMCs after lactate treatment. n = 4 per group. (**G**) Representative images of Alizarin red and Von Kossa staining of VSMCs after the indicated treatments and CM exposure for another 7 days. Scale bars, 5 mm (upper panels), 100 μm (lower panels). Statistical significance was assessed using t-test (A) and one-way ANOVA followed by

Dunnett's test (D, E, F). All values are presented as mean ± SD. *$P < 0.05$, **$P < 0.01$, ***$P < 0.001$, and ****$P < 0.0001$.

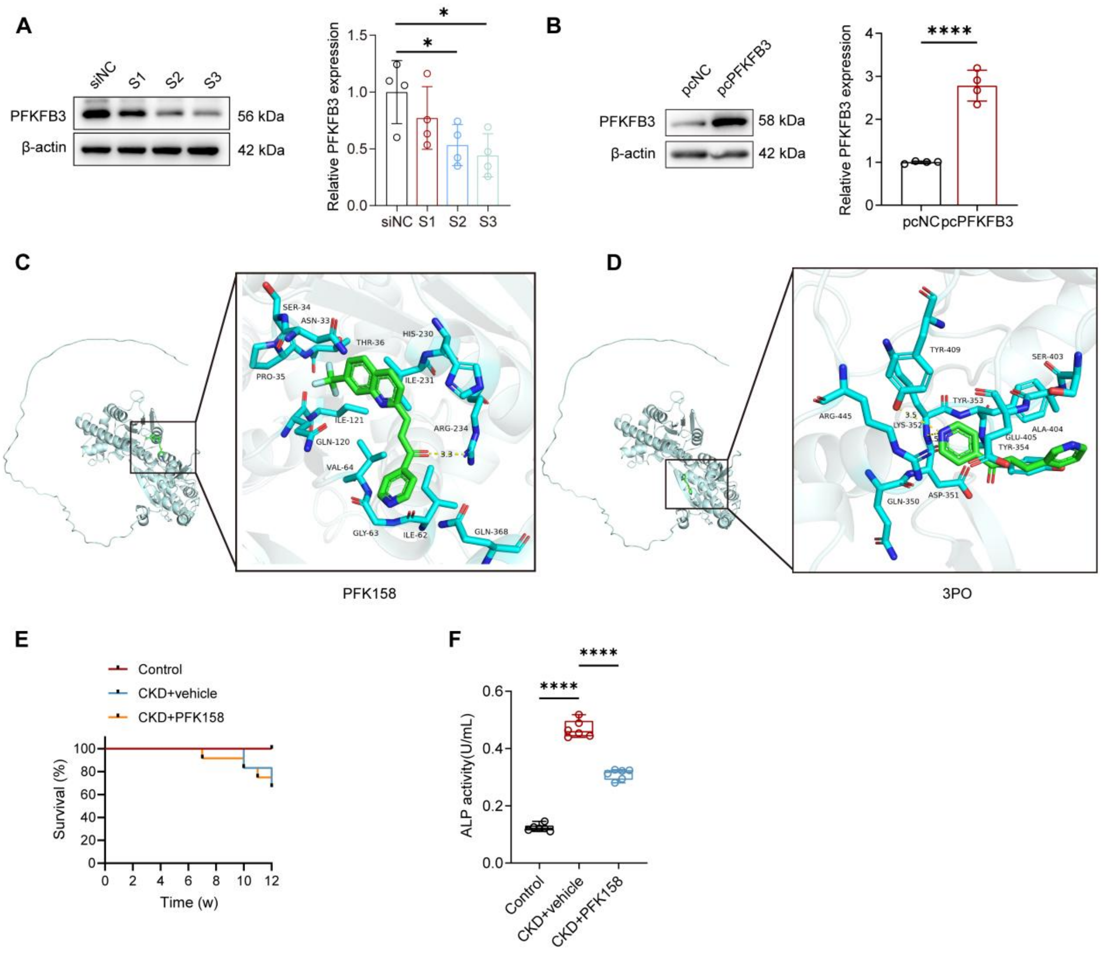

Supplemental Figure S8. OTUB2 exerts procalcific effects through PFKFB3 upregulation. (**A**) Western blot analysis identifying the PFKFB3 depletion in VSMCs. n = 4 per group. (**B**) Western blot analysis identifying the PFKFB3 overexpression in VSMCs. n = 4 per group. (**C**) Molecular docking analysis of PFK158 with PFKFB3. (**D**) Molecular docking analysis of 3PO with PFKFB3. (**E**) The survival curves of mice in each group. (**F**) ALP activity assay. n = 6 per group. Statistical significance was assessed using t-test (B) and one-way ANOVA followed by Dunnett's test (A, F). All values are presented as mean ± SD. *$P < 0.05$, **$P < 0.01$, ***$P < 0.001$, and ****$P < 0.0001$.

Supplemental tables

Table S1. The relative siRNAs in this work

Gene name	Relative sequences
OTUB2 siRNA S1 sense	GAGAGAUCCUCAAGUUCAATT
OTUB2 siRNA S1 antisense	UUGAACUUGAGGAUCUCUCTT
OTUB2 siRNA S2 sense	CGGUUUAUCUGCUCUAUAATT
OTUB2 siRNA S2 antisense	UUCAUCAGAAGAACCAGCCTT
OTUB2 siRNA S3 sense	CAGAGUUCAACAAACUCAATT
OTUB2 siRNA S3 antisense	UUGAGUUUGUUGAACUCUGTT
YAP siRNA S1 sense	CGGUUGAAACAACAGGAAUUATT
YAP siRNA S1 antisense	UAAUUCCUGUUGUUUCAACCGTT
YAP siRNA S2 sense	UGAGAACAAUGACAACCAAUATT
YAP siRNA S2 antisense	UAUUGGUUGUCAUUGUUCUCATT
YAP siRNA S3 sense	GAAGCGCUGAGUUCCGAAAUCTT
YAP siRNA S3 antisense	GAUUUCGGAACUCAGCGCUUCTT
PFKFB3 siRNA S1 sense	CCGCUCAUGAGACGCAAUATT
PFKFB3 siRNA S1 antisense	UAUUGCGUCUCAUGAGCGGTT
PFKFB3 siRNA S2 sense	GGAAUUAGAGCGCCAAGAGAATT
PFKFB3 siRNA S2 antisense	UUCUCUUGGCGCUCUAAUUCCTT
PFKFB3 siRNA S3 sense	GCCCUGAGCAAGUUCGUAGAATT
PFKFB3 siRNA S3 antisense	UUCUACGAACUUGCUCAGGGCTT

Table S2. The antibodies used in this study

Antibodies	Item number	Usage
Anti-Runx2	Abcam # ab236639	WB 1:1000; IHC/IF 1:200
Anti-BMP2	Immunoway # YT5651	WB 1:1000; IHC/IF 1:200
Anti-α-SMA	Abcam # ab7817	WB 1:1000; IHC/IF 1:200
Anti-OTUB2	Immunoway # YT3475	WB 1:1000; IHC/IF 1:200
Anti-YAP	Proteintech # 13584-1-AP	WB 1:5000; IHC/IF 1:200
Anti-active YAP	Abcam # ab205270	WB 1:1000
Anti-PFKFB3	Abcam # ab181861	WB 1:1000; IHC/IF 1:100
Anti-Ub K48	Abcam # ab140601	WB 1:1000
Anti-Ub K63	Abcam # ab179434	WB 1:1000
Anti-Flag	Abmart # M20008	WB 1:5000
Anti-HA	Abmart # M20013	WB 1:5000
Anti-GAPDH	Proteintech # 10494-1-AP	WB 1:5000
Anti-β-actin	Proteintech # 66009-1-Ig	WB 1:5000
Anti-Histone3	Proteintech # 17168-1-AP	WB 1:5000
Anti-α-tubulin	Proteintech # 11224-1-AP	WB 1:5000
Anti-LATS1	Abcam # ab243656	WB 1:1000
Anti-mouse-Alexa Flu-488	Abways # ab0142	IF: 1:200
Anti-rabbit-Alexa Flu-594	Abways # ab0151	IF: 1:200
IgG	Proteintech # 30000-0-AP	IP: 2μl for 1mg protein

Table S3. The primers of relative genes in this work

Gene Name	Primers
mGAPDH Forward(5'- 3')	CAGTGGCAAAGTGGAGATTGTTG
mGAPDH Reverse(5'- 3')	TCGCTCCTGGAAGATGGTGAT
mOTUB2 Forward(5'- 3')	AACCGAGCTGACTTCTTCCGAC
mOTUB2 Reverse(5'- 3')	GCAGAGCAATGTTGAGTGCCTG
mOTUB1 Forward(5'- 3')	GCTGGAACTCTCAGTCCTGTAC
mOTUB1 Reverse(5'- 3')	CGGTAGAAGCAGTTGCCATCAG
mYAP Forward(5'- 3')	CCAGACGACTTCCTCAACAGTG
mYAP Reverse(5'- 3')	GCATCTCCTTCCAGTGTGCCAA
mCTGF Forward(5'- 3')	TGCGAAGCTGACCTGGAGGAAA
mCTGF Reverse(5'- 3')	CCGCAGAACTTAGCCCTGTATG
mANKRD1 Forward(5'- 3')	GCTTAGAAGGACACTTGGCGATC
mANKRD1 Reverse(5'- 3')	GACATCTGCGTTTCCTCCACGA
mAREG Forward(5'- 3')	GCAGATACATCGAGAACCTGGAG
mAREG Reverse(5'- 3')	CCTTGTCATCCTCGCTGTGAGT
mGLUT1 Forward(5'- 3')	GCTTCTCCAACTGGACCTCAAAC
mGLUT1 Reverse(5'- 3')	ACGAGGAGCACCGTGAAGATGA
mHK2 Forward(5'- 3')	CCCTGTGAAGATGTTGCCCACT
mHK2 Reverse(5'- 3')	CCTTCGCTTGCCATTACGCACG
mLDHA Forward(5'- 3')	ACGCAGACAAGGAGCAGTGGAA
mLDHA Reverse(5'- 3')	ATGCTCTCAGCCAAGTCTGCCA

mPKM2 Forward(5'- 3')	CAGAGAAGGTCTTCCTGGCTCA
mPKM2 Reverse(5'- 3')	GCCACATCACTGCCTTCAGCAC
mPFKFB3 Forward(5'- 3')	TCATCGAGTCGGTCTGTGACGA
mPFKFB3 Reverse(5'- 3')	CATGGCTTCTGCTGAGTTGCAG
mβ- actin Forward(5'- 3')	CATTGCTGACAGGATGCAGAAGG
mβ- actin Reverse(5'- 3')	TGCTGGAAGGTGGACAGTGAGG

Table S4. Baseline characteristics of the study patients with or without vascular calcification.

Characteristics	Non-calcification (n = 8)	Calcification (n = 8)	*P* value
Age, years	64.25±11.16	61.50±12.85	0.65
Male, n (%)	4 (50.00%)	7 (87.50%)	0.28
SBP, mmHg	150.90±24.29	131.80±10.25	0.06
DBP, mmHg	81.13±20.11	80.13±9.172	0.90
Hemoglobin, g/L	90.75±16.71	96.63±27.22	0.61
eGFR, mL/min/1.73 m^2	6.56±3.16	5.50 (4.00-10.10)	0.86
BUN, mmol/L	23.03±10.38	23.69±6.786	0.88
CREA, µmol/L	711.10±402.00	813.40±337.10	0.59
Phosphate, mmol/L	1.53±0.46	1.73±0.25	0.28
Calcium, mmol/L	2.29±0.22	2.22±0.30	0.59
Kalium, mmol/L	4.16±0.61	4.06±0.31	0.69
Total cholesterol, mmol/L	3.18±0.73	3.19±0.85	0.97
Triglycerides, mmol/L	1.39 (0.71-3.15)	0.85 (0.75-2.51)	0.33

HDL cholesterol, mmol/L	0.95±0.51	0.89±0.16	0.78
LDL cholesterol, mmol/L	1.85±0.36	1.88±0.59	0.92
ALB, g/L	36.91±3.36	33.49±6.71	0.22
ALT, U/L	10.70±3.98	11.55 (7.75-13.63)	0.90
AST, U/L	16.76±2.44	15.35 (11.53-20.38)	0.49

Values are expressed as median (25th to 75th quartiles) or mean ± SD for continuous variables and n (%) for categorical variables, respectively. SBP, systolic blood pressure; DBP, diastolic blood pressure; eGFR, estimated glomerular filtration rate; BUN, blood urea nitrogen; CREA, creatinine; HDL, high-density lipoprotein; LDL, low-density lipoprotein. ALB, albumin; ALT, alanine transaminase; AST, aspartate aminotransferase.

Table S5. Bodyweight and plasma biochemical parameters of adenine diet-induced CKD mice with OTUB2 deficiency or overexpression.

Parameters	Control	CKD+AAV-NC	CKD+AAV-shOTUB2	CKD+AAV-OTUB2 OE
Body weight (g)	28.71±1.30	14.01±1.68*	15.14±1.36*	14.95±0.83*
Creatinine (μM)	18.91±8.64	56.72±7.46*	63.49±18.30*	70.20±9.65*
BUN (mM)	5.92±3.52	60.16±10.76*	50.07±972*	51.97±10.42*
Phosphorus (mM)	2.95±0.50	6.16±0.86*	6.20±2.17*	6.54±1.67*
Calcium (mM)	2.29±0.27	2.25±0.14	2.33±0.03	2.45±0.14

Statistical significance was assessed using one-way ANOVA followed by Dunnett's test. Values are means ± SD (n = 6 per group). $*P < 0.05$ vs. Control.

Table S6. Bodyweight and plasma biochemical parameters of adenine diet-induced CKD mice treated with vehicle or OTUB2-IN-1.

Parameters	Control	CKD+vehicle	CKD+OTUB2-IN-1
Body weight (g)	28.93±1.66	14.18±1.77*	14.27±1.81*
Creatinine (μM)	25.36±5.79	62.03±11.45*	65.12±20.59*
BUN (mM)	7.19±4.99	77.13±12.83*	80.96±15.15*
Phosphorus (mM)	2.10±0.39	4.08±0.50*	3.50±1.08*
Calcium (mM)	2.43±0.11	2.40±0.06	2.49±0.11

Statistical significance was assessed using one-way ANOVA followed by Dunnett's test. Values are means ± SD (n = 6 per group). *$P < 0.05$ vs. Control.

Table S7. Bodyweight and plasma biochemical parameters of adenine diet-induced CKD mice with YAP deficiency or VTP treatment after OTUB2 overexpression.

Parameters	Control	CKD+ AAV-NC	CKD+AAV-OTUB2 OE	CKD+ AAV-shYAP	CKD+AAV-OTUB2 OE+shYAP	CKD+VTP	CKD+AAV-OTUB2 OE+VTP
Body weight (g)	29.43±1.35	15.33±1.19*	14.74±1.07*	14.97±0.97*	14.90±0.99*	14.70±1.13*	14.65±1.33*
Creatinine (μM)	21.15±3.89	53.41±6.50*	52.70±8.31*	48.38±11.20*	54.05±3.57*	55.66±6.94*	51.46±6.94*
BUN (mM)	5.37±1.21	54.49±9.05*	54.44±12.49*	47.93±8.67*	47.92±15.82*	55.39±15.78*	55.96±15.17*
Phosphorus (mM)	3.15±0.68	7.46±1.06*	8.18±1.32*	7.07±1.70*	8.10±1.57*	7.44±3.01*	7.50±1.91*

Calcium (mM)	2.59±0.07	2.48±0.20	2.37±0.22	2.39±0.57	2.27±0.17	2.66±0.32	2.62±0.25

Statistical significance was assessed using one-way ANOVA followed by Dunnett's test. Values are means ± SD (n = 6 per group). *$P < 0.05$ vs. Control.

Table S8. Bodyweight and plasma biochemical parameters of adenine diet-induced CKD mice treated with vehicle or PFK158.

Parameters	Control	CKD+vehicle	CKD+PFK158
Body weight (g)	29.33±1.21	14.87±1.04*	14.87±0.87*
Creatinine (μM)	23.82±5.70	62.46±14.17*	51.43±4.43*
BUN (mM)	18.41±3.57	88.28±8.98*	80.13±18.10*
Phosphorus (mM)	3.52±0.48	5.41±0.28*	6.11±0.72*
Calcium (mM)	2.20±0.20	2.11±0.19	2.28±0.21

Statistical significance was assessed using one-way ANOVA followed by Dunnett's test. Values are means ± SD (n = 6 per group). *$P < 0.05$ vs. Control.
